# Supplementary material for: Food-insecure pregnant women in South Africa: a cross-sectional exploration of maternal depression as a mediator of violence and trauma risk factors
Source: BMJ Open. 2019 Mar 13;9(3):e018277. doi: 10.1136/bmjopen-2017-018277 (PMC6429723; doi:10.1136/bmjopen-2017-018277)
Supplement: Supplementary file 1 [file bmjopen-2017-018277supp001.pdf]

Supplemental Table 1. Sensitivity analysis: comparison of key sociodemographic variables between participants included in and excluded from the current study.

	**Included sample**	**Excluded sample**	**p-value**
Number of mothers	992	233	
Clinic			
Mbekweni	549 (55)	131 (56)	
TC Newman	443 (45)	102 (44)	0.808
Race			
Black	548 (55)	132 (57)	
Coloured	443 (45)	101 (43)	0.708
Maternal Income			
<R1,000/month	767 (77)	168 (78)	
R1000-R5000/month	212 (21)	44 (20)	0.954
R5000-R10,000/month	12 (1)	3 (1)	
Receive social assistance	491 (49)	92 (40)	0.005
Maternal education			
Some secondary	613 (62)	127 (55)	
Completed Secondary	379 (38)	103 (45)	0.066
Median number of children in household	1	1	0.911
Married/cohabiting	399 (40)	92 (40)	0.828
Employed	254 (26)	67 (29)	0.429
