# Supplementary material for: Food-insecure pregnant women in South Africa: a cross-sectional exploration of maternal depression as a mediator of violence and trauma risk factors
Source: BMJ Open. 2019 Mar 13;9(3):e018277. doi: 10.1136/bmjopen-2017-018277 (PMC6429723; doi:10.1136/bmjopen-2017-018277)
Supplement: Author's manuscript [file bmjopen-2017-018277.draft_revisions.pdf]

BMJ Open is committed to open peer review. As part of this commitment we make the peer review history of every article we publish publicly available.

When an article is published we post the peer reviewers' comments and the authors' responses online. We also post the versions of the paper that were used during peer review. These are the versions that the peer review comments apply to.

The versions of the paper that follow are the versions that were submitted during the peer review process. They are not the versions of record or the final published versions. They should not be cited or distributed as the published version of this manuscript.

BMJ Open is an open access journal and the full, final, typeset and author-corrected version of record of the manuscript is available on our site with no access controls, subscription charges or pay-per-view fees (http://bmjopen.bmj.com).

If you have any questions on BMJ Open's open peer review process please email info.bmjopen@bmj.com

BMJ Open

Maternal depression as a mediator for psychosocial risk factors of food insecurity among pregnant women in a South African setting.

Journal:	*BMJ Open*
Manuscript ID	bmjopen-2017-018277
Article Type:	Research
Date Submitted by the Author:	23-Apr-2018
Complete List of Authors:	Barnett, Whitney; University of Cape Town, Department of Paediatrics and Child Health, Red Cross War Memorial Children's Hospital; South African Medical Research Council Unit on Child & Adolescent Health<br>Pellowski, Jennifer; The Warren Alpert Medical School of Brown University, Department of Psychiatry and Human Behavior<br>Kuo, Caroline; Brown University, Department of Behavioral and Social Sciences and Center for Alcohol and Addiction Studies<br>Koen, Nastassja; University of Cape Town, Department of Psychiatry and Mental Health; South African Medical Research Council Unit on Risk and Resilience in Mental Disorders<br>Donald, Kirsten; University of Cape Town, Division of Developmental Paediatrics, Department of Paediatrics & Child Health, Red Cross War Memorial Children's Hospital<br>Zar, Heather; University of Cape Town, Department of Paediatrics and Child Health, Red Cross War Memorial Children's Hospital; South African Medical Research Council, Unit on Child and Adolescent Health<br>Stein, Dan; University of Cape Town, Department of Psychiatry and Mental Health; South African Medical Research Council Unit on Risk and Resilience in Mental Disorders
Keywords:	food insecurity, maternal mental health, intimate partner violence, depression mediation

SCHOLARONE™
Manuscripts

Maternal depression as a mediator for psychosocial risk factors of food insecurity among pregnant women in a South African setting.

Authors: Whitney Barnett[1], Jennifer Pellowski[2], Caroline Kuo[3,4], Nastassja Koen[4,5], Kirsty A Donald[6], Heather J. Zar[1], Dan J. Stein[4,5]

[1] Department of Paediatrics and Child Health, Red Cross War Memorial Children's Hospital, and Medical Research Council Unit on Child & Adolescent Health, University of Cape Town, South Africa, [2] Department of Psychiatry and Human Behavior, The Warren Alpert Medical School of Brown University, Providence, Rhode Island, USA [3]Department of Behavioral and Social Sciences and Center for Alcohol and Addiction Studies, Brown University, Providence Rhode Island, USA [4]Department of Psychiatry and Mental Health, University of Cape Town, South Africa [5]South African Medical Research Council Unit on Risk and Resilience in Mental Disorders, Cape Town, South Africa [6]Division of Developmental Paediatrics, Department of Paediatrics & Child Health, Red Cross War Memorial Children's Hospital, University of Cape Town, Cape Town, South Africa

Corresponding author:
Whitney Barnett
Department of Paediatrics and Child Health
Unit B11, REACH Center
Red Cross War Memorial Children's Hospital
Cape Town, South Africa
barnett.whitney@gmail.com
Tel: +27 (0) 790432604

Key words: food insecurity, maternal mental health, intimate partner violence, depression mediation

Word count: 3,805

For peer review only

Abstract

Purpose: Food insecurity during pregnancy is associated with lower infant birthweight and children from food insecure households have increased rates of developmental delay and poorer general health. Better understanding of psychological risk factors for food insecurity, and how they interact is crucial, given the long-term health implications for maternal and child wellbeing. This study investigated the association between psychological risk factors and food insecurity among pregnant women in a South African birth cohort, the Drakenstein Child Health Study (DCHS) and whether maternal depression mediates these relationships.

Methods: We investigated psychosocial predictors of food insecurity using multivariate regression. Mediation analyses were conducted to explore whether depression mediated the relationship between intimate partner violence (IPV) and food insecurity as well as between childhood trauma and food insecurity including disaggregation by two study communities.

Results: Among 992 pregnant women (median age 26 years), there were high rates of IPV (emotional IPV, 27%; physical IPV, 22%; sexual IPV, 7%), depression (24%), and childhood trauma (34%). In multivariate cross sectional analysis, emotional IPV (AOR 1.605; p-value<0.05), depression (AOR 1.052; p-value<0.01) and childhood trauma (AOR 1.484; p-value<0.05) predicted food insecurity. In mediation models including both communities, depression partially mediated the relationship between emotional IPV and food insecurity as well as physical IPV and food insecurity; depression partially mediated the relationship between childhood trauma and food insecurity. Differing degrees of mediation were found when applied to communities separately.

Conclusions: Antenatal maternal mental health problems were highly prevalent and associated with food insecurity. These results highlight the importance of addressing maternal depression, childhood trauma and IPV in relation to food security; screening services during antenatal care may offer an opportunity to identify and intervene in these issues. Community level differences in risk and in mediation analyses indicate that contextual tailoring of interventions may be an important consideration.

Strengths & Limitations of this study:

- There are few studies investigating depression as a mediator in relationships between subtypes of maternal intimate partner violence or childhood trauma and food insecurity during pregnancy.
- This study extends existing related research to an African population with a large sample size.
- The findings of the current study may inform improved screening strategies in future by highlighting psychosocial risk factors linked to food insecurity during pregnancy.
- The current study included cross-sectional data; therefore further research is needed to assess the direction of causality, and if differences exist by trimester and postpartum.

For peer review only

Background

Food insecurity is the lack of nutritionally adequate and safe food or a limited ability to acquire necessary food in socially acceptable ways.[1] The Food and Agriculture Organization (FAO) estimates that 842 million people (12%) worldwide were unable to meet their dietary requirements in 2011-2013. This is equal to 1 in 8 people with insufficient food for a healthy life, the majority from low- and middle-income countries (LMICs).[2] Studies have shown a link between food insecurity and poor pregnancy outcomes, including low birth weight,[3] gestational diabetes, and pregnancy complications.[4] In addition, young children in food insecure households have poorer general health[5-7] increased probability of being hospitalized,[6,8] lower levels of parent-child attachment,[9] and increased developmental delays.[9-11] Chronic hunger in childhood has also been linked to a higher likelihood of chronic medical conditions and asthma.[12,13] Pregnant women may be particularly vulnerable to food insecurity due to increased nutrient demands and the inability to continue working, leading to financial strain.

Maternal mental health disorders are prevalent in LMICs and have been linked to food insecurity. Examining maternal mental health characteristics in relation to food insecurity in LMICs is important, particularly in the context of high proportions of maternal headed and single parent households, and given that food security is a managed process such that family members have some control over how they cope with food insecurity and who within the family experiences it. This ability to manage the effects of food insecurity may be adversely affected by maternal mental health.[14]

Maternal mental health problems such as depression,[15] stressful life events and trauma[16-19] are associated with food insecurity as well as poorer pregnancy outcomes such as low infant birth weight,[20] impaired foetal[21] and infant growth and nutritional status[3,22] as well as poorer infant cognitive development.[8,23] Of particular interest is intimate partner violence (IPV) and maternal childhood trauma, which have been linked to food insecurity. For example, research has found maternal childhood trauma to be significantly associated with food insecurity[24] and that mothers of severely hungry school-aged children are more likely to have a lifetime post traumatic stress disorder (PTSD) diagnosis.[25] Although the relationship between maternal mental health and food insecurity has been explored, few studies have investigated depression as a mediator in the relationship between other psychosocial risk factors and food insecurity. Further, the majority of studies have focused on high-income countries[18,26-28] or have used small sample sizes to explore associations.[29,30] We explore depression as a mediator in the relationship between IPV as well as childhood trauma and food insecurity in a LMIC context where risk factors such as poor maternal mental health, IPV, and childhood trauma are high. This extends the evidence base to geographic regions where these issues are highly prevalent but the relationships between these variables are poorly understood and little investigated.

Given the long-term health implications of food insecurity for maternal and child wellbeing[31-33] understanding how risk factors for poor child health outcomes interact is critical to inform public policy to address the most urgent modifiable risk factors. Data from this study have shown links between psychosocial risk factors and food insecurity during pregnancy;[34] this paper builds upon that research by investigating the mediational effects of maternal depression on the relationship between emotional, physical and sexual IPV as well as maternal childhood trauma and food insecurity. Addressing food insecurity during pregnancy offers an opportunity to link antenatal care with nutritional programs and

For peer review only - http://bmjopen.bmj.com/site/about/guidelines.xhtml

manage associated mental health risk factors at a time when those risk factors impact not only the mother's safety and well being but also infant outcomes after birth.

Methods

The Drakenstein Child Health Study (DCHS) is a multidisciplinary population-based birth cohort study located in a peri-urban area, 60km outside of Cape Town, South Africa.[35] It is a stable, low socioeconomic community comprising approximately 200 000 people, characterized by a high prevalence of a range of health risk factors such as single-parent households, depression, childhood trauma, IPV and poverty.[36] Maternal psychosocial health, food security and sociodemographics were measured at an antenatal visit between 28 to 32 weeks' gestation, following enrollment.

Participants

Pregnant women were enrolled from March 2012 to March 2015 and followed through childbirth. Women were enrolled in their second trimester, between 20 to 28 weeks' gestation at two public sector primary health care clinics, one serving a predominantly mixed ethnicity population (TC Newman) and the other serving a predominantly Black African population (Mbekweni). Eligibility criteria were 1) attendance at one of the two study clinics 2) being at least 18 years of age and 3) intending to remain in the study area for at least 1 year.

Measures

Maternal sociodemographics and mental health was measured using validated questionnaires administered by trained study staff at an antenatal visit at 28 to 32 weeks' gestation. Mental health assessments included measures of intimate partner violence, depression, childhood trauma, stressful life events and psychological distress. The Intimate Partner Violence (IPV) Questionnaire used in this study was adapted from the WHO multi-country study[37] and the Women's Health Study in Zimbabwe [38]. Participants were dichotomized into exposed or unexposed for having experienced emotional, physical or sexual IPV in the past 12 months. The Edinburgh Postnatal Depression Scale (EPDS)[39] was used to measure depression; this scale has been validated for use with pregnant women and in a South African population.[40,41] The EPDS consists of 10 items referring to the past 7 days with each item assessed on a scale from 0 to 3. A total score was obtained by summing responses for all items and was included as a continuous score, with higher scores indicating more severe depressive symptoms. The Childhood Trauma Questionnaire[43] Short-Form was used to assess abuse and neglect experienced as a child. Each item was responded to on a 5 point scale ranging from *1=never true* to *5=very often true*. Continuous scores were used with a total possible range from 28 to 140. The Modified World Mental Health Life Events Questionnaire, adapted based on items used in the South African Stress and Health Study (SASH) in South Africa,[44] to measure stressful or negative life events in the past year. Items were scored according to whether or not the event was experienced, *0=no*, *1=yes*. Individual items were then summed to create a total score, ranging from 0 to 17, with higher scores indicating greater exposure to stressful life events. The SRQ-20 is a WHO-endorsed measure of psychological distress.[45] The SRQ-20 consists of 20 items, which assess non-psychotic symptoms, including symptoms of depressive and anxiety disorders, scored according to whether or not the symptom was present, *0=no, 1=yes*. Individual items are summed to generate a total score ranging from 0 to 20, with higher scores indicating higher levels of psychological distress.[46]

For peer review only - http://bmjopen.bmj.com/site/about/guidelines.xhtml

Sociodemographic variables including mother-reported paternal characteristics, household factors and maternal demographics were collected using an interviewer-administered questionnaire adapted from items used in the SASH Study.[44] Socioeconomic status (SES) was measured based on a composite score of asset ownership, household income, employment and education.[44] Perceived food insecurity was assessed using an adapted version of the U.S. Department of Agriculture (USDA) Household Food Security Scale[1] which captures food hardship due to financial constraints (smaller meals, skipped meals, hunger). Five items were used and an affirmative response to two or more items was coded as being food insecure.

Ethics

Ethical approval was obtained from the Faculty of Health Sciences Research Ethics Committee, University of Cape Town and the Provincial Research committee. Mothers gave written informed consent at enrolment.

Patient and public involvement

Prior to study initiation, local stakeholders (Department of Health staff and managers) were involved in the planning of the parent study, the Drakenstein Child Health birth cohort study. Patients and public were not involved in conceptualisation or analysis of the specific aims reported in the current study, however, study findings are routinely fedback to the study community.

Statistical Analysis

All data were analysed using SPSS. Univariate logistic regression analyses were conducted to determine the bivariate relationship between food insecurity and demographic and psychosocial predictors. Odds ratios (ORs) with p-values were calculated to determine the strength of these associations. A hierarchical multivariate logistic regression analysis was conducted to independently evaluate IPV exposure on food insecurity prior to the addition of previously investigated psychosocial factors, while controlling for demographic variables. Block 1 included community, maternal income, and maternal education. Block 2 included recent experiences of emotional, physical, and sexual IPV. Finally, Block 3 included depression, childhood trauma, stressful life events, and psychological distress. To determine whether depression played a mediating role on the relationship between IPV and food insecurity, meditational analyses were conducted using PROCESS macro.[47] Model number 4 was used and indirect effects were bootstrapped using 1000 samples. Beta coefficients and standard errors are reported for all paths and 95% confidence intervals are reported for the indirect effects. Models were conducted for the full sample and then for each community individually. This process was replicated for depression as a mediator of the relationship between childhood trauma and food insecurity. Mediation models controlled for community, maternal income, maternal education, social grants, number of children in the household and HIV status; childhood trauma was controlled for in all IPV mediation models and emotional, physical, and sexual IPV was controlled for in the childhood trauma mediation models.

Results

A total of 1225 pregnant women were enrolled between March 2012 and February 2015; of these, 992 women had complete data and were included in the analysis. Missing data resulted from non-attendance at one of two visits. Detailed baseline demographic characteristics, stratified by recruitment site, are presented in Table 1. The median age of participants was 26.6 years [standard deviation (SD) 5.8]. The sample was characterized by low SES - 77% of mothers had a monthly income of less than R1,000

For peer review only - http://bmjopen.bmj.com/site/about/guidelines.xhtml

(approximately 100 USD), 49% of mothers were receiving social assistance, 26% reported being employed and 38% completed secondary education (high school). A minority of mothers (40%) were married or with a partner. HIV prevalence and SES quartiles were significantly different between clinics as were the majority of psychosocial variables. Mothers at TC Newman were significantly more likely to have experienced emotional and sexual past-year IPV as well as childhood trauma and stressful life events. Co-occurrence of mental health issues was prevalent, though more so at TC Newman. Overall, 12% of mothers had both depression and IPV, 13% depression and childhood trauma and 16% childhood trauma and any form of IPV.

Table 1. Maternal demographic and psychological variables

	Overall	TC Newman	Mbekweni	X^2	P-value
Number of mothers	992	443	549		
Mean age of mother (SD)	26.6 (5.8)	25.7 (5.4)	27.3 (5.9)	-4.543*	p<0.001
Race					
Black	548 (55)	6 (1)	542 (99)	943.05	<0.001
Coloured	443 (45)	437 (99)	6 (1)		
SES Quartiles					
Lowest SES	258 (26)	81 (18)	177 (32)	37.27	<0.001
Low-moderate SES	261 (26)	117 (26)	144 (26)		
Moderate-high SES	242 (24)	109 (25)	133 (24)		
Highest SES	231 (23)	136 (31)	95 (17)		
Maternal Income					
<R1,000/month	767 (77)	330 (74)	437 (80)	7.86	0.049
R1000-R5000/month	212 (21)	103 (23)	109 (20)		
R5000-R10,000/month	12 (1)	9 (2)	3 (1)		
Receive social assistance	491 (49)	221 (50)	270 (49)	0.085	0.798
Maternal education					
Some secondary	613 (62)	266 (60)	347 (63)	1.037	0.308
Completed Secondary	379 (38)	177 (40)	202 (37)		
Median number of children in household	1	1	1	22.191	0.002
Married/cohabiting	399 (40)	200 (45)	199 (36)	10.064	0.018
Employed	254 (26)	132 (30)	122 (22)	7.439	0.007
Maternal HIV	216 (22)	17 (4)	199 (36)	151.195	<0.001
Psychosocial risk factors**					
Past year IPV					
Emotional IPV	266 (27)	155 (35)	111 (20)	27.26	<0.001
Physical IPV	216 (22)	106 (24)	110 (20)	2.18	0.14
Sexual IPV	68 (7)	49 (11)	19 (3)	22.179	<0.001
Depression	242 (24)	112 (25)	130 (24)	0.341	0.559
Childhood trauma	335 (34)	179 (40)	156 (28)	15.761	<0.001
Psychological Distress	208 (21)	109 (25)	99 (18)	6.39	0.011
Stressful Life Events	449 (45)	265 (60)	184 (34)	68.467	<0.001
Co-occurrence of psychosocial risk factors					
Depression & any IPV	122 (12)	68 (15)	54 (10)	6.911	0.009
Depression & childhood trauma	124 (13)	76 (17)	48 (9)	15.864	<0.001
Childhood trauma & any IPV	154 (16)	101 (23)	53 (10)	32.304	<0.001

**t-test*
***Psychological risk factors listed where above threshold; IPV above threshold = score of >1 within each subtype (mothers experiencing more than an isolated incidence in past year); Depression above threshold = score ≥ 13; Childhood trauma above threshold where score > 36; Psychological Distress dichotomized into low and high risk categories where high risk = score ≥8; Stressful life events presented where greater than 1.*

Univariate analysis

In univariate analysis (Table 2), antenatal food insecurity was significantly more likely among participants from Mbekweni (OR 5.68; p-value <0.001), those who had not completed secondary school

For peer review only - http://bmjopen.bmj.com/site/about/guidelines.xhtml

(OR 0.43; p-value <0.001), mothers with lower income levels (OR 0.39; p-value <0.001), mothers who had experienced emotional IPV (OR 1.45; p-value <0.05) or physical IPV (OR 1.85; p-value <0.001) in the past twelve months and mothers with higher levels of antenatal depression (OR 1.09; p-value <0.001), childhood trauma (OR 1.49; p-value <0.01) and psychological distress (OR 1.42; p-value <0.05).

Table 2. Hierarchical logistic regression of variables associated with food insecurity.

	Univariate Regressions		Block 1		Block 2		Block 3	
Variables	Unadjusted OR	p-value	Adjusted OR	p-value	Adjusted OR	p-value	Adjusted OR	p-value
Demographic variables								
Community	5.679	***	6.015	***	7.751	***	7.876	***
Maternal Income	0.394	***	0.421	***	0.422	***	0.449	***
Maternal Education	0.427	***	0.421	***	0.431	***	0.452	***
Intimate Partner Violence								
Emotional IPV	1.445	*			1.707	*	1.605	*
Physical IPV	1.853	***			1.433	0.102	1.315	0.220
Sexual IPV	1.624	0.059			1.882	0.05	1.463	0.253
Psychological Variables ****								
Depression (EPDS)	1.087	***					1.052	**
Childhood Trauma	1.491	**					1.484	*
Stressful Life Events	0.780	0.074					0.937	0.708
Psychological Distress	1.416	*					1.138	0.547
			Block X2(df)	p-value	Block X2(df)	p-value	Block X2(df)	p-value
			188.93 (3)	***	29.76 (3)	***	20.04 (4)	***

*p<0.05, **p<0.01, ***p<0.001
**** Psychological variables were included as continuous scores in regression analyses.

Hierarchical regression

A hierarchical logistic regression was done to investigate the additive impact of risk factor groups on food security (Table 2). Throughout all blocks, community, maternal education and maternal income remained significantly associated with food insecurity. In block 2 among additional IPV risk factors, adjusting for maternal sociodemographic factors and community, only physical IPV was a significant predictor. In the final model (block 3), which incorporated all psychological variables, IPV and demographic variables, mothers from Mbekweni were almost eight times (OR 7.88; p-value <0.001) as likely as TC Newman mothers to experience antenatal food insecurity. Mothers who completed secondary school were 55% less likely to experience food insecurity (OR 0.45; p-value <0.001) compared to mothers who did not complete secondary school. Similarly, mothers with higher incomes were 55% (OR 0.45; p-value <0.001) less likely to experience food insecurity. Mothers who experienced emotional IPV in the past twelve months were 60% more likely (OR 1.60; p-value <0.05), mothers with higher depression scores on EPDS were 5% more likely (OR 1.05; p-value <0.01) and mothers with a history of childhood trauma were 48% more likely (OR 1.48; p-value <0.05) than mothers without these psychological risk factors to experience food insecurity.

Depression as a mediator of the relationship between IPV and food insecurity

In mediation models including both communities (figure 1), depression partially mediated the relationship between emotional IPV and food insecurity (direct effect p-value = 0.0001; indirect effect = 0.16, 95% CI 0.07, 0.29) and partially mediated the relationship between physical IPV and food insecurity (direct effect p-value =0.001; indirect effect = 0.17, 95% CI 0.07, 0.28). Sexual IPV was not tested in a mediation

For peer review only - http://bmjopen.bmj.com/site/about/guidelines.xhtml

model because the bivariate relationship between sexual IPV and food insecurity was not significant (OR 1.62; p=0.06). Mediation models were split by community due to the high significance of recruitment community as an independent predictor of food insecurity; when split by community differing degrees of mediation were found. At TC Newman (figure 2), depression fully mediated the relationship between emotional IPV and food insecurity (direct effect p-value = 0.13; indirect effect = 0.24, 95% CI 0.05, 0.50) as well as between physical IPV and food insecurity (indirect effect = 0.23, 95% CI 0.06, 0.46). At Mbekweni (figure 3), depression partially mediated the relationship between emotional IPV and food insecurity (direct effect p-value = 0.0004; indirect effect = 0.11, 95% CI 0.03, 0.25) and partially mediated the relationship between physical IPV and food insecurity (direct effect p-value = 0.008; indirect effect = 0.13, 95% CI 0.04, 0.29).

Depression as a mediator of the relationship between childhood trauma and food insecurity

In mediation models including both communities (figure 4), depression partially mediated the relationship between childhood trauma and food insecurity (direct effect p-value = 0.025; indirect effect = 0.127, 95% CI 0.05, 0.23). These mediation models were also split by community, due to the high significance of community as a risk factor for food insecurity. When applying mediation models to childhood trauma at TC Newman, depression did not mediate the relationship between childhood trauma and food insecurity (direct effect p-value = 0.0009; indirect effect 0.12, 95% CI -0.12, 0.35). Additionally, at Mbekweni, depression did not mediate the relationship between childhood trauma and food insecurity (direct effect p-value = 0.63; indirect effect 0.04, 95% CI -0.02, 0.13).

Discussion

Our goal was to investigate the association between IPV and other psychosocial risk factors with food insecurity during pregnancy, as well as to investigate maternal depression as a mediator for these relationships in a LMIC country, South Africa. We found significant effects of emotional IPV and maternal childhood trauma on antenatal food insecurity, after adjusting for community, maternal income and education. Mothers experiencing emotional IPV or with a history of childhood trauma were 60% and 52% more likely, respectively, to live in food insecure households while pregnant. Though previous studies have investigated links between IPV or childhood trauma and food insecurity, the majority were in high-income countries,[18,26,27] where programs and support networks addressing these risk factors are more robust. Others utilized relatively small sample sizes[29,30] and the vast majority do not differentiate between subtypes of IPV,[18] which may differ in their impact on food insecurity and maternal mental health, employment capabilities or maternal household managerial capacity.

Hernandez and colleagues found that IPV was a significant predictor of food insecurity and that this was mediated by depression.[48] However, this US-based study did not find significant associations between subtypes of IPV and food insecurity - only a composite measure of IPV was found to be significant. Our research extends this to a LMIC setting and found that sub-types of IPV differentially predicted food insecurity, with emotional IPV the only significant predictor in the final model. While mediation models split by site found a mediational effect of depression on this relationship at both clinics; emotional IPV did maintain a direct effect on food insecurity at Mbekweni. This may be an important distinction when planning effective interventions that consider community contexts; qualitative research has found that women feel emotionally abusive acts are more devastating than physical violence.[49] Emotional IPV, therefore, may be a critical and often overlooked risk factor for food insecurity; and may manifest

For peer review only - http://bmjopen.bmj.com/site/about/guidelines.xhtml

differently in a LMIC setting compared to a high-income setting possibly linked to traditional gender norms affecting women's sense of power and identity and therefore ability to manage household resources.[50]

Maternal childhood trauma also emerged as a critical risk factor for food insecurity during pregnancy. Sun and colleagues investigated this link in a large US-based study.[24 29,30] These authors reported that childhood trauma was linked to food insecurity during pregnancy,[34] but did not include maternal depression as a potential mediator in this relationship. While other high-income country studies have also investigated this association, these have been limited by small sample sizes (n=44, n=31). To our knowledge, our study is the first to investigate this link in a LMIC. Childhood trauma measured by family instability, violence exposure at a young age, food insecurity in childhood and neglect is associated with many of the known risk factors of current food insecurity such as lower levels of education, employment and poor mental health outcomes in adulthood.[51] This highlights a critical link between childhood experiences and adult outcomes and the intergenerational effects of trauma. In the context of maternal mental health, this may be particularly relevant as maternal hardship and stress may increase the likelihood of a traumatic childhood for their offspring. As noted by Sun and colleagues, there is an intergenerational transmission of disadvantage, which highlights the need for a multi-faceted approach to address food insecurity. Our findings reveal important intergenerational risks for food insecurity via maternal childhood exposure to violence and suggest that future research is needed to understand how intergenerational transmission of trauma occurs between mothers and children and what can be done to break this cycle. In high-prevalence settings in particular, intervention programs should offer more than nutrition support and should include trauma-informed mental health services to reduce the transmission of trauma from one generation to the next.[52]

The co-occurrence of psychosocial risk factors was high (12-16%) in our study sample. In order to better understand how these risk factors influence one another, we investigated depression as a mediator in the these relationships. In overall models depression partially mediated the relationship between emotional and physical IPV and food insecurity. Notably, the degree to which depression mediated this relationship differed between clinics. Depression fully mediated the relationship between emotional and physical IPV and food insecurity at TC Newman, but only partial mediation was found at Mbekweni. This highlights depression as important in the pathway through which IPV affects food insecurity at TC Newman. However, at Mbekweni, though depression also exacerbates this relationship, there may be other factors that explain the significant relationship between IPV and food insecurity. While maternal income was controlled for in mediation models, SES quartiles indicate that Mbekweni mothers are economically worse off than TC Newman mothers. This may be impeding the process of food management at Mbekweni, especially in the context of IPV. In overall models investigating depression as a mediator for the relationship between childhood trauma and food security, partial mediation was again seen; however, no mediation was found when models were split by community. At TC Newman, childhood trauma maintained a significant direct effect on food insecurity. It may be that social support networks are more robust at Mbekweni, thus mitigating the downstream effects of childhood trauma in that community. Further research is needed to understand why depression mediated the effects of childhood trauma on food insecurity in overall models whereas this effect did not persist when models were split by community.

For peer review only - http://bmjopen.bmj.com/site/about/guidelines.xhtml

Strengths & Limitations

The inclusion criteria for the parent study were broad to ensure generalizability. However, recruitment was done during antenatal care visits, so mothers who did not present for antenatal care or who presented in their third trimester were excluded, which may affect overall generalizability. In addition, approximately 200 mothers who were enrolled in the study were not included in the analysis due to incomplete data. While it is possible that this subset of mothers is at higher risk for many of the factors investigated; there were not significant differences in key factors investigated for mothers included versus those excluded in the current study. As the current study included cross-sectional data, we cannot assert the direction of causality. While a strength of this study was its investigation of the individual impact of different subtypes of IPV, too few mothers experienced or reported sexual IPV to allow this to be investigated fully. Finally, as all variables used were based on self-report, mothers may have underreported both risk factors and food security due to social desirability bias. Despite these limitations, the current study provides a novel quantitative analysis with a large sample size conducted in a LMIC. Our findings corroborate previous research on risk factors for food insecurity; and build on unpublished data in this cohort by investigating subtypes of IPV as well as maternal depression as a mediator for both childhood trauma and IPV.

Conclusions

Addressing depression during pregnancy through screening and referral services may help to alleviate the negative impact of IPV, childhood trauma and depression on food security. Both IPV victimization and experiencing childhood trauma may lead to depressive symptoms in mothers, impacting their household managerial skills by decreasing motivation to obtain food, to find and hold employment and through decreased physical and cognitive functioning. Our findings highlight the importance of comprehensive programs aimed at nutrition support or food security, also addressing multiple concurrent risk factors to reduce food insecurity and alleviate its negative impact on child health. However, notably, mediation models indicate that the effects of multiple maternal mental health issues are experienced differently at the two communities in this cohort highlighting the importance of program and policy efforts targeted to specific community profiles.

Acknowledgements

We thank the study staff in Paarl, the study data team and lab teams, the clinical and administrative staff of the Western Cape Government Health Department at Paarl Hospital and at the clinics for support of the study. We acknowledge the advice from members of the study International Advisory Board and thank our collaborators. We thank the families and children who participated in this study.

Declaration of interest

The authors have no conflicts of interest to declare.

Funding

The study was funded by the Bill and Melinda Gates Foundation [OPP 1017641]; additional support from the National Institutes of Health (H3Africa 1U01AI110466-01A1), the South African MRC, the National Research Foundation, South Africa, the UK Royal Society, Newton Advanced Fellowship.

Author contributions:

WB & JP conceptualized the analysis and wrote the first draft of the manuscript. HJZ is principal

For peer review only - http://bmjopen.bmj.com/site/about/guidelines.xhtml

investigator of the parent study; DS leads the psychosocial study aspects; NK and KD are co-investigators and contributed to the study design and implementation. JP conducted the data analysis. CK, JP and DS provided critical inputs on the manuscript. All authors read and approved the final manuscript.

Data sharing statement: No additional data are available.

For peer review only

References

1. Bickel G, Nord M, Price C, Hamilton W, Cook J, Guide to Measuring Household Food Security, Revised 2000. U.S. Department of Agriculture, Food and Nutrition Service, Alexandria VA. March, 2000.
2. Food and Agriculture Organization, The State of Food Insecurity in the World 2013. The multiple dimensions of food security. Rome, FAO. 2013.
3. Borders AE, Grobman WA, Amsden LB, Holl JL. Chronic stress and low birth weight neonates in a low-income population of women. *Obstetrics and gynecology* 2007; **109**(2 Pt 1): 331-8.
4. Laraia BA, Siega-Riz AM, Gundersen C. Household food insecurity is associated with self-reported pregravid weight status, gestational weight gain, and pregnancy complications. *Journal of the American Dietetic Association* 2010; **110**(5): 692-701.
5. Cook JT, Black M, Chilton M, et al. Are food insecurity's health impacts underestimated in the U.S. population? Marginal food security also predicts adverse health outcomes in young U.S. children and mothers. *Advances in nutrition (Bethesda, Md)* 2013; **4**(1): 51-61.
6. Cook JT, Frank DA, Levenson SM, et al. Child food insecurity increases risks posed by household food insecurity to young children's health. *The Journal of nutrition* 2006; **136**(4): 1073-6.
7. Bronte-Tinkew J, Zaslow M, Capps R, Horowitz A, McNamara M. Food insecurity works through depression, parenting, and infant feeding to influence overweight and health in toddlers. *The Journal of nutrition* 2007; **137**(9): 2160-5.
8. Cook JT, Frank DA, Berkowitz C, et al. Food insecurity is associated with adverse health outcomes among human infants and toddlers. *The Journal of nutrition* 2004; **134**(6): 1432-8.
9. Zaslow M, Bronte-Tinkew J, Capps R, Horowitz A, Moore KA, Weinstein D. Food security during infancy: implications for attachment and mental proficiency in toddlerhood. *Maternal and child health journal* 2009; **13**(1): 66-80.
10. Hernandez DC, Jacknowitz A. Transient, but not persistent, adult food insecurity influences toddler development. *The Journal of nutrition* 2009; **139**(8): 1517-24.
11. Rose-Jacobs R, Black MM, Casey PH, et al. Household food insecurity: associations with at-risk infant and toddler development. *Pediatrics* 2008; **121**(1): 65-72.
12. Kirkpatrick SI, McIntyre L, Potestio ML. Child hunger and long-term adverse consequences for health. *Archives of pediatrics & adolescent medicine* 2010; **164**(8): 754-62.
13. Mangini LD, Hayward MD, Dong YQ, Forman MR. Household Food Insecurity is Associated with Childhood Asthma. *The Journal of nutrition* 2015; **145**(12): 2756-64.
14. Radimer KL, Harvey PW, Green A, Orrell E. Compliance with dietary goals in a Queensland community. *Australian journal of public health* 1992; **16**(3): 277-81.
15. Ivers LC, Cullen KA. Food insecurity: special considerations for women. *The American journal of clinical nutrition* 2011; **94**(6): 1740S-4S.
16. Davison KM, Kaplan BJ. Food insecurity in adults with mood disorders: prevalence estimates and associations with nutritional and psychological health. *Annals of general psychiatry* 2015; **14**: 21.
17. Martin MS, Maddocks E, Chen Y, Gilman SE, Colman I. Food insecurity and mental illness: disproportionate impacts in the context of perceived stress and social isolation. *Public health* 2016.
18. Melchior M, Caspi A, Howard LM, et al. Mental health context of food insecurity: a representative cohort of families with young children. *Pediatrics* 2009; **124**(4): e564-72.
19. Sorsdahl K, Slopen N, Siefert K, Seedat S, Stein DJ, Williams DR. Household food insufficiency and mental health in South Africa. *Journal of epidemiology and community health* 2011; **65**(5): 426-31.
20. Koen N, Wyatt GE, Williams JK, et al. Intimate partner violence: associations with low infant birthweight in a South African birth cohort. *Metabolic brain disease* 2014; **29**(2): 281-99.

For peer review only

21. Brittain K, Myer L, Koen N, et al. Risk Factors for Antenatal Depression and Associations with Infant Birth Outcomes: Results From a South African Birth Cohort Study. *Paediatric and perinatal epidemiology* 2015.
22. Ross J, Hanlon C, Medhin G, et al. Perinatal mental distress and infant morbidity in Ethiopia: a cohort study. *Archives of disease in childhood Fetal and neonatal edition* 2011; **96**(1): F59-64.
23. Rahman A, Harrington R, Bunn J. Can maternal depression increase infant risk of illness and growth impairment in developing countries? *Child: care, health and development* 2002; **28**(1): 51-6.
24. Sun J, Knowles M, Patel F, Frank DA, Heeren TC, Chilton M. Childhood Adversity and Adult Reports of Food Insecurity Among Households With Children. *American journal of preventive medicine* 2015.
25. Weinreb L, Wehler C, Perloff J, et al. Hunger: Its impact on children's health and mental health. *Pediatrics* 2002;110;e41.
26. Wehler C, Weinreb LF, Huntington N, et al. Risk and protective factors for adult and child hunger among low-income housed and homeless female-headed families. *American journal of public health* 2004; **94**(1): 109-15.
27. Kathree T, Selohilwe OM, Bhana A, Petersen I. Perceptions of postnatal depression and health care needs in a South African sample: the "mental" in maternal health care. *BMC women's health* 2014; **14**: 140.
28. Weaver LJ, Hadley C. Moving beyond hunger and nutrition: a systematic review of the evidence linking food insecurity and mental health in developing countries. *Ecology of food and nutrition* 2009; **48**(4): 263-84.
29. Chilton MM, Rabinowich JR, Woolf NH. Very low food security in the USA is linked with exposure to violence. *Public health nutrition* 2014; **17**(1): 73-82.
30. Chilton M, Knowles M, Rabinowich J, Arnold KT. The relationship between childhood adversity and food insecurity: 'It's like a bird nesting in your head'. *Public health nutrition* 2015; **18**(14): 2643-53.
31. Maggi S, Irwin LJ, Siddiqi A, Hertzman C. The social determinants of early child development: an overview. *Journal of paediatrics and child health* 2010; **46**(11): 627-35.
32. Taylor SE. Mechanisms linking early life stress to adult health outcomes. *Proceedings of the National Academy of Sciences of the United States of America* 2010; **107**(19): 8507-12.
33. Kingston D, Tough S. Prenatal and postnatal maternal mental health and school-age child development: a systematic review. *Maternal and child health journal* 2014; **18**(7): 1728-41.
34. Pellowski J, Barnett W, Kuo CC, Koen N, Zar HJ, Stein DJ. Predicting food insecurity during pregnancy among women in a South African birth cohort study – an ecological approach. *Public health nutr* 2015. Under review..
35. Zar HJ, Barnett W, Myer L, Stein DJ, Nicol MP. Investigating the early-life determinants of illness in Africa: the Drakenstein Child Health Study. *Thorax* 2014.
36. Stein DJ, Koen N, Donald KA, et al. Investigating the psychosocial determinants of child health in Africa: The Drakenstein Child Health Study. *Journal of neuroscience methods* 2015; **252**: 27-35.
37. Jewkes R. Intimate partner violence: causes and prevention. *Lancet* 2002; **359**(9315): 1423-9.
38. Shamu S, Abrahams N, Temmerman M, Musekiwa A, Zarowsky C. A systematic review of African studies on intimate partner violence against pregnant women: prevalence and risk factors. *PloS one* 2011; **6**(3): e17591.
39. Cox JL, Holden JM, Sagovsky R. Detection of postnatal depression. Development of the 10-item Edinburgh Postnatal Depression Scale. *The British journal of psychiatry : the journal of mental science* 1987; **150**: 782-6.
40. Lawrie TA, Hofmeyr GJ, de Jager M, Berk M. Validation of the Edinburgh Postnatal Depression Scale on a cohort of South African women. *South African medical journal = Suid-Afrikaanse tydskrif vir geneeskunde* 1998; **88**(10): 1340-4.

For peer review only

41. Tsai AC, Scott JA, Hung KJ, et al. Reliability and validity of instruments for assessing perinatal depression in African settings: systematic review and meta-analysis. *PloS one* 2013; **8**(12): e82521.
42. Murray D, Cox JL. Screening for depression during pregnancy with the Edinburgh Depression Scale (EPDS). *J Reprod Infant Psychol* 1990; 8:99-107.
43. Bernstein DP, Fink L, Handelsman L, et al. Initial reliability and validity of a new retrospective measure of child abuse and neglect. *The American journal of psychiatry* 1994; **151**(8): 1132-6.
44. Myer L, Stein DJ, Grimsrud A, Seedat S, Williams DR. Social determinants of psychological distress in a nationally-representative sample of South African adults. *Social science & medicine (1982)* 2008; **66**(8): 1828-40.
45. Beusenberg M, Orley J. A user's guide to the self reporting questionnaire (SRQ). Division of Mental Health. Geneva: World Health Organization: 1994.
46. Ventevogel P, De Vries G, Scholte WF, et al. Properties of the Hopkins Symptom Checklist-25 (HSCL-25) and the Self-Reporting Questionnaire (SRQ-20) as screening instruments used in primary care in Afghanistan. *Social psychiatry and psychiatric epidemiology* 2007; **42**(4): 328-35.
47. Hayes AF. Introduction to Mediation, Moderation, and Conditional Process Analysis: A Regression-Based Approach. . *Guilford Press* 2013.
48. Hernandez DC, Marshall A, Mineo C. Maternal depression mediates the association between intimate partner violence and food insecurity. *Journal of women's health* 2014; **23**(1): 29-37.
49. World Health Organization. WHO multi-country study on women's health and domestic violence against women: summary report of initial results on prevalence, health outcomes and women's responses. Geneva, WHO, 2012.
50. Department of Women, Children & People with Disabilities (DWCPD). Stop violence against women in South Africa. Cape Town, DWCPD, 2013.
51. Messina N, Grella C. Childhood trauma and women's health outcomes in a California prison population. *American journal of public health* 2006; **96**(10): 1842-8.
52. Sun YY, Li Y, Wali B, et al. Prophylactic Edaravone Prevents Transient Hypoxic-Ischemic Brain Injury: Implications for Perioperative Neuroprotection. *Stroke; a journal of cerebral circulation* 2015; **46**(7): 1947-55.
53. Dewing S, Tomlinson M, le Roux IM, Chopra M, Tsai AC. Food insecurity and its association with co-occurring postnatal depression, hazardous drinking, and suicidality among women in peri-urban South Africa. *Journal of affective disorders* 2013; **150**(2): 460-5.

Figure legends

Fig 1. Mediation models for both communities investigating depression as a mediator; depression **partially** mediates the relationship between emotional IPV and food insecurity as well as the relationship between physical IPV and food insecurity. Covariates included in the model are: community, maternal income, maternal education, social grants, number of children in the household, HIV status and childhood trauma.

Fig 2. Mediation models for TC Newman investigating depression as a mediator; depression **fully** mediates the relationship between emotional IPV and food insecurity as well as physical IPV and food insecurity. Covariates included in the model are: community, maternal income, maternal education, social grants, number of children in the household, HIV status and childhood trauma.

Fig 3. Mediation models at Mbekweni investigating depression as a mediator; depression **partially** mediates the relationship between emotional IPV and food insecurity as well as the relationship between

For peer review only

physical IPV and food insecurity. Covariates included in the model are: community, maternal income, maternal education, social grants, number of children in the household, HIV status and childhood trauma.

Fig 4. Mediation models for both communities, TC Newman and Mbekweni investigating depression as a mediator for childhood trauma and food insecurity. Depression **partially** mediated the overall relationship between childhood trauma and food insecurity, but **did not mediate** the relationship at TC Newman and **did not** mediate the relationship at Mbekweni. Covariates included in the model are: community, maternal income, maternal education, social grants, number of children in the household, HIV status and emotional, physical and sexual forms of IPV.

For peer review only

For peer review only - http://bmjopen.bmj.com/site/about/guidelines.xhtml

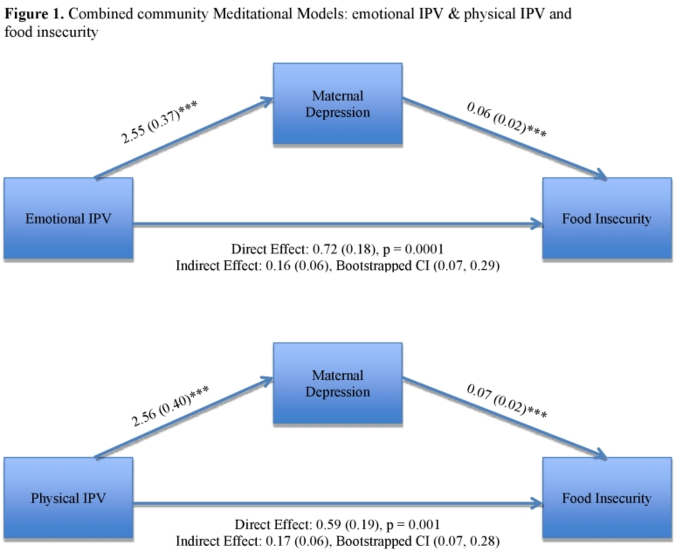

Fig 1. Mediation models for both communities investigating depression as a mediator; depression partially mediates the relationship between emotional IPV and food insecurity as well as the relationship between physical IPV and food insecurity. Covariates included in the model are: community, maternal income, maternal education, social grants, number of children in the household, HIV status and childhood trauma.

209x297mm (300 x 300 DPI)

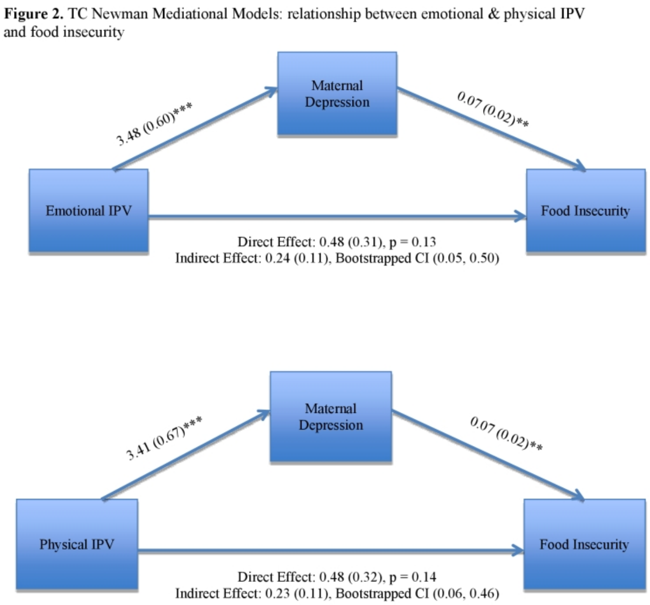

Fig 2. Mediation models for TC Newman investigating depression as a mediator; depression fully mediates the relationship between emotional IPV and food insecurity as well as physical IPV and food insecurity. Covariates included in the model are: community, maternal income, maternal education, social grants, number of children in the household, HIV status and childhood trauma.

209x297mm (300 x 300 DPI)

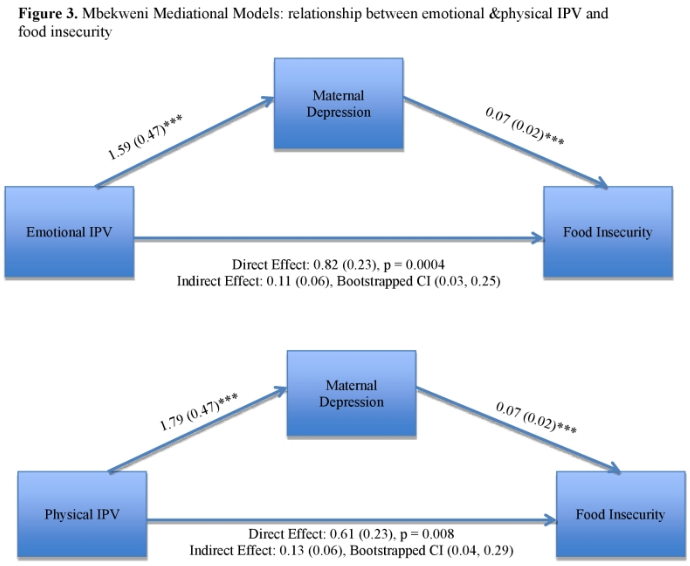

Fig 3. Mediation models at Mbekweni investigating depression as a mediator; depression partially mediates the relationship between emotional IPV and food insecurity as well as the relationship between physical IPV and food insecurity. Covariates included in the model are: community, maternal income, maternal education, social grants, number of children in the household, HIV status and childhood trauma.

209x297mm (300 x 300 DPI)

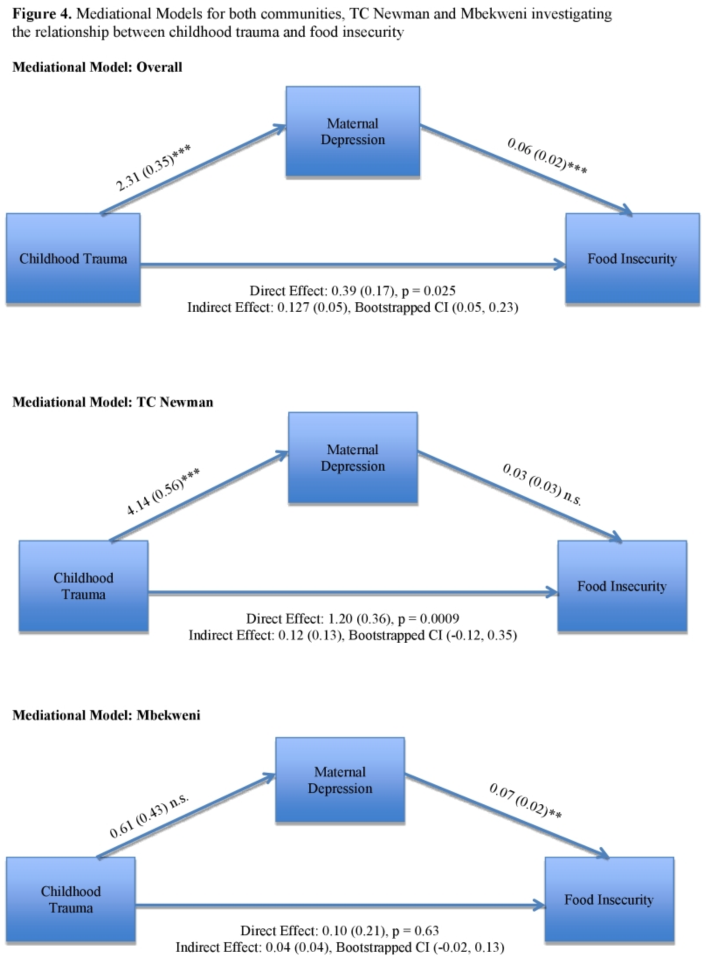

Fig 4. Mediation models for both communities, TC Newman and Mbekweni investigating depression as a mediator for childhood trauma and food insecurity. Depression partially mediated the overall relationship between childhood trauma and food insecurity, but did not mediate the relationship at TC Newman and did not mediate the relationship at Mbekweni. Covariates included in the model are: community, maternal income, maternal education, social grants, number of children in the household, HIV status and emotional, physical and sexual forms of IPV.

209x297mm (300 x 300 DPI)

STROBE Statement

Checklist of items that should be included in reports of observational studies

Section/Topic	Item No	Recommendation	Reported on Page No
**Title and abstract**	1	(*a*) Indicate the study's design with a commonly used term in the title or the abstract	2
		(*b*) Provide in the abstract an informative and balanced summary of what was done and what was found	2
**Introduction**			
Background/rationale	2	Explain the scientific background and rationale for the investigation being reported	3
Objectives	3	State specific objectives, including any prespecified hypotheses	3
**Methods**			
Study design	4	Present key elements of study design early in the paper	4
Setting	5	Describe the setting, locations, and relevant dates, including periods of recruitment, exposure, follow-up, and data collection	4
Participants	6	*Cross-sectional study*—Give the eligibility criteria, and the sources and methods of selection of participants	4,5
Variables	7	Clearly define all outcomes, exposures, predictors, potential confounders, and effect modifiers. Give diagnostic criteria, if applicable	4,5
Data sources/measurement	8*	For each variable of interest, give sources of data and details of methods of assessment (measurement). Describe comparability of assessment methods if there is more than one group	4,5
Bias	9	Describe any efforts to address potential sources of bias	10
Study size	10	Explain how the study size was arrived at	5
Quantitative variables	11	Explain how quantitative variables were handled in the analyses. If applicable, describe which groupings were chosen and why	4,5
Statistical methods	12	(*a*) Describe all statistical methods, including those used to control for confounding	5
		(*b*) Describe any methods used to examine subgroups and interactions	5,7
		(*c*) Explain how missing data were addressed	5
		(*d*) *Cohort study*—If applicable, explain how loss to follow-up was addressed<br>*Case-control study*—If applicable, explain how matching of cases and controls was addressed<br>*Cross-sectional study*—If applicable, describe analytical methods taking account of sampling strategy	n/a
		(*e*) Describe any sensitivity analyses	10

For peer review only - http://bmjopen.bmj.com/site/about/guidelines.xhtml

Section/Topic	Item No	Recommendation	Reported on Page No
**Results**			
Participants	13*	(a) Report numbers of individuals at each stage of study—eg numbers potentially eligible, examined for eligibility, confirmed eligible, included in the study, completing follow-up, and analysed	5
		(b) Give reasons for non-participation at each stage	
		(c) Consider use of a flow diagram	n/a
Descriptive data	14*	(a) Give characteristics of study participants (eg demographic, clinical, social) and information on exposures and potential confounders	6
		(b) Indicate number of participants with missing data for each variable of interest	5
		(c) *Cohort study*—Summarise follow-up time (eg, average and total amount)	n/a
Outcome data	15*	*Cohort study*—Report numbers of outcome events or summary measures over time	n/a
		Case-control study—Report numbers in each exposure category, or summary measures of exposure	
		Cross-sectional study—Report numbers of outcome events or summary measures	6
Main results	16	(*a*) Give unadjusted estimates and, if applicable, confounder-adjusted estimates and their precision (eg, 95% confidence interval). Make clear which confounders were adjusted for and why they were included	7
		(*b*) Report category boundaries when continuous variables were categorized	6
		(*c*) If relevant, consider translating estimates of relative risk into absolute risk for a meaningful time period	n/a
Other analyses	17	Report other analyses done—eg analyses of subgroups and interactions, and sensitivity analyses	8,10
**Discussion**			
Key results	18	Summarise key results with reference to study objectives	8
Limitations	19	Discuss limitations of the study, taking into account sources of potential bias or imprecision. Discuss both direction and magnitude of any potential bias	10
Interpretation	20	Give a cautious overall interpretation of results considering objectives, limitations, multiplicity of analyses, results from similar studies, and other relevant evidence	8,9,10
Generalisability	21	Discuss the generalisability (external validity) of the study results	10
**Other Information**			
Funding	22	Give the source of funding and the role of the funders for the present study and, if applicable, for the original study on which the present article is based	10

**Give information separately for cases and controls in case-control studies and, if applicable, for exposed and unexposed groups in cohort and cross-sectional studies.*

Note: An Explanation and Elaboration article discusses each checklist item and gives methodological background and published examples of transparent reporting. The STROBE checklist is best used in conjunction with this article (freely available on the Web sites of PLoS Medicine at http://www.plosmedicine.org/, Annals of Internal Medicine at http://www.annals.org/, and Epidemiology at http://www.epidem.com/). Information on the STROBE Initiative is available at www.strobe-statement.org.

For peer review only - http://bmjopen.bmj.com/site/about/guidelines.xhtml

BMJ Open

Food insecure pregnant women in South Africa: Maternal depression mediates violence and trauma risk factors.

Journal:	*BMJ Open*
Manuscript ID	bmjopen-2017-018277.R1
Article Type:	Research
Date Submitted by the Author:	26-Sep-2018
Complete List of Authors:	Barnett, Whitney; University of Cape Town, Department of Paediatrics and Child Health, Red Cross War Memorial Children's Hospital; South African Medical Research Council Unit on Child & Adolescent Health<br>Pellowski, Jennifer; Brown University School of Public Health, Department of Behavioral and Social Sciences and International Health Institute<br>Kuo, Caroline; Brown University, Department of Behavioral and Social Sciences and Center for Alcohol and Addiction Studies<br>Koen, Nastassja; University of Cape Town, Department of Psychiatry and Mental Health; South African Medical Research Council Unit on Risk and Resilience in Mental Disorders<br>Donald, Kirsten; University of Cape Town, Division of Developmental Paediatrics, Department of Paediatrics & Child Health, Red Cross War Memorial Children's Hospital<br>Zar, Heather; University of Cape Town, Department of Paediatrics and Child Health, Red Cross War Memorial Children's Hospital; South African Medical Research Council, Unit on Child and Adolescent Health<br>Stein, Dan; University of Cape Town, Department of Psychiatry and Mental Health; South African Medical Research Council Unit on Risk and Resilience in Mental Disorders
**Primary Subject Heading**:	Public health
Secondary Subject Heading:	Nutrition and metabolism, Mental health
Keywords:	food insecurity, maternal mental health, intimate partner violence, depression mediation

SCHOLARONE™
Manuscripts

Food insecure pregnant women in South Africa: Maternal depression mediates violence and trauma risk factors.

Authors: Whitney Barnett[1], Jennifer Pellowski[2], Caroline Kuo[3,4], Nastassja Koen[4,5], Kirsty A Donald[6], Heather J. Zar[1], Dan J. Stein[4,5]

[1] Department of Paediatrics and Child Health, Red Cross War Memorial Children's Hospital, and Medical Research Council Unit on Child & Adolescent Health, University of Cape Town, South Africa,
[2] Department of Behavioral and Social Sciences and International Health Institute, Brown University School of Public Health, Providence, Rhode Island, USA [3]Department of Behavioral and Social Sciences and Center for Alcohol and Addiction Studies, Brown University, Providence Rhode Island, USA
[4]Department of Psychiatry and Mental Health, University of Cape Town, South Africa [5]South African Medical Research Council Unit on Risk and Resilience in Mental Disorders, Cape Town, South Africa
[6]Division of Developmental Paediatrics, Department of Paediatrics & Child Health, Red Cross War Memorial Children's Hospital, University of Cape Town, Cape Town, South Africa

Corresponding author:
Whitney Barnett
Department of Paediatrics and Child Health
Unit B11, REACH Center
Red Cross War Memorial Children's Hospital
Cape Town, South Africa
barnett.whitney@gmail.com
Tel: +27 (0) 790432604

Key words: food insecurity, maternal mental health, intimate partner violence, depression, mediation

Word count: 4,462

For peer review only

Abstract

Objectives: Better understanding of psychosocial risk factors for food insecurity (FI) during pregnancy, and how they interact is crucial, given long-term health implications for maternal and child health. We investigated the association between maternal childhood trauma as well as intimate partner violence (IPV) and FI among pregnant women in South Africa, in the Drakenstein Child Health Study, and whether maternal depression mediates these relationships.

Setting: Two primary care clinics in Paarl, South Africa.

Participants: 992 pregnant women; inclusion criteria were clinic attendance and remaining in area for at least 1 year; women were excluded if a minor.

Methods: We examined psychosocial predictors of FI using multivariate regression. Mediation analyses investigated whether depression mediated the relationship between IPV and FI as well as between childhood trauma and FI, including disaggregation by two study communities. FI was assessed using an adapted US Department of Agriculture food security scale; households were coded as food insecure where 2 of 5 affirmative responses were recorded.

Results: Among 992 pregnant women there were high rates of IPV (7-27%), depression (24%), and childhood trauma (34%). In multivariate cross-sectional analysis, emotional IPV (aOR 1.60; 95% CI 1.04,2.46), depression (aOR 1.05; 95% CI 1.01,1.08) and childhood trauma (aOR 1.52; 95% CI 1.08,2.15) predicted FI. In mediation models depression partially mediated the relationship between emotional IPV and FI as well as physical IPV and FI; depression partially mediated the relationship between childhood trauma and FI. Differing degrees of mediation were found when applied to communities.

Conclusions: Antenatal maternal depression, IPV and childhood trauma were highly prevalent and associated with FI. Depression, IPV and trauma screening services should be considered within routine antenatal care and may offer an opportunity to identify and intervene. Community level differences in risk and in mediation analyses indicate that contextual tailoring of interventions may be important.

Strengths & Limitations of this study:

- There are few studies investigating depression as a mediator in relationships between subtypes of maternal intimate partner violence or childhood trauma and food insecurity during pregnancy.
- This study extends existing related research to a low resource African population with a large sample size.
- The current study was a cross-sectional analysis; therefore further research is needed to assess the direction of causality, and if differences exist by trimester and postpartum.

For peer review only - http://bmjopen.bmj.com/site/about/guidelines.xhtml

Background

Food insecurity is the lack of nutritionally adequate and safe food or a limited ability to acquire necessary food in socially acceptable ways.[1] The Food and Agriculture Organization (FAO) estimates that 689 million people worldwide (1 in 10) suffer from severe food insecurity (2014-2016); Africa has the highest prevalence of severe food insecurity (27.4%), almost four times the prevalence of other regions.[2] Studies have shown a link between food insecurity and poor pregnancy outcomes, including low birth weight,[3] gestational diabetes, and pregnancy complications.[4] In addition, young children in food insecure households have poorer general health[5-7] increased probability of being hospitalized,[6,8] lower levels of parent-child attachment,[9] and increased developmental delays.[9-11] Chronic hunger in childhood has also been linked to a higher likelihood of chronic medical conditions, such asthma, heart conditions, kidney disease or allergies.[12] Pregnant women may be particularly vulnerable to food insecurity due to increased nutrient demands and the inability to continue working, leading to financial strain.

Maternal mental health disorders are prevalent in low and middle-income countries (LMICs). Maternal mental health problems such as depression[13] and psychosocial risk factors such as stressful life events, intimate partner violence and trauma[14-17] are associated with food insecurity as well as poorer pregnancy outcomes such as low infant birth weight,[18] impaired foetal[19] and infant growth and nutritional status[3,20] as well as poorer infant cognitive development.[8,21] Although the relationship between maternal trauma or violence exposures as well as mental health and food insecurity has been explored, few studies have investigated depression as a mediator in the relationship between other psychosocial risk factors (e.g. violence or trauma) and food insecurity. Sun and colleagues, in a large US based study, found maternal childhood trauma to be linked to food insecurity during pregnancy and that depression mediated this relationship.[22] Others in the US have found similar links between childhood trauma and food insecurity, but have not investigated mental health pathways.[23] In another US based study, IPV was found to be a significant predictor of food insecurity, mediated by depression.[24] However, this study did not find differential associations between sub-types of IPV (emotional, physical and sexual), though others have.[25] The majority of studies have focused on high-income countries[14,16,22,24,26] or have used small sample sizes to explore associations.[14,23] The current study aims to extend previous research to a LMIC context and to analyse multiple exposures, maternal trauma, IPV and stressful events, which are often co-occuring and have a higher prevalence in LMIC settings in a large study sample.

Examining maternal psychosocial risk factors and mental health characteristics in relation to food insecurity in LMICs is important. Particularly in the context of high proportions of maternal headed and single parent households and given the high prevalence of maternal psychosocial risk factors, especially during pregnancy, when exposures can adversely affect both maternal and child long-term health. Food security is a managed process such that family members have some control over how they cope with food insecurity and who within the family experiences it.[26,27] This ability to manage the effects of food insecurity may be adversely affected by maternal psychosocial risk factors and maternal mental health.[16] Further, community level factors such as differences in stigma to accessing care, gender norms affecting agency or education levels for women, may have significant differential effects within communities. This community context may be important to understand how to best address key risk factors for food insecurity and to inform design of effective interventions.

We therefore aim to explore associations between maternal psychosocial risk factors or mental health and

For peer review only - http://bmjopen.bmj.com/site/about/guidelines.xhtml

food insecurity, disaggregated by two communities with different risk profiles and community level factors. We investigate whether depression acts as a mediator in the relationship between IPV or childhood trauma and food insecurity in a LMIC context,(see figure 1: conceptual framework). This extends the evidence base to geographic regions where these issues are highly prevalent but the relationships between these variables are poorly understood and rarely investigated.

Given the long-term health implications of food insecurity for child development as well as maternal and child mental and physical health,[12-15] understanding how risk factors for poor child health outcomes interact is critical to inform public policy to address the most urgent modifiable risk factors. Previous published findings from this cohort have shown links between psychosocial risk factors and food insecurity during pregnancy;[28] this paper builds upon that research by investigating the mediational effects of maternal depression on the relationship between emotional, physical and sexual IPV and food insecurity as well as maternal childhood trauma and food insecurity. Addressing food insecurity during pregnancy offers an opportunity to link antenatal care with nutritional programs and manage associated mental health risk factors at a time when those risk factors impact not only the mother's safety and well being but also infant outcomes after birth.

Methods

The Drakenstein Child Health Study (DCHS) is a multidisciplinary population-based birth cohort study located in a peri-urban area, 60 km outside of Cape Town, South Africa. It is a low socioeconomic community comprising approximately 200 000 people predominantly of mixed-ancestry (62.5%; 13.5% caucasian; 22.7% Black African).[29] The district is characterized by a high prevalence of a range of health risk factors such as single-parent households, depression, childhood trauma, IPV, poverty, low levels of education (27.4% completing secondary school) and high unemployment (17.6%). The DCHS is a longitudinal cohort study following mother-child dyads through early childhood.[30,31] The current analysis utilises data from two antenatal visits: maternal psychosocial health and food security were measured at an antenatal visit between 28 to 32 weeks' gestation; sociodemographics were measured at the enrolment visit, at 20-28 weeks' gestation.

Participants

Pregnant women were enrolled from March 2012 to March 2015. Women were enrolled in their second trimester, between 20 to 28 weeks' gestation at two public sector primary health care clinics, one serving a predominantly mixed-ancestry population (TC Newman) and the other serving a predominantly Black African population (Mbekweni). Inclusion criteria were 1) attendance at one of the two study clinics 2) and 2) intending to remain in the study area for at least 1 year. Mothers were excluded if they were under 18 years of age at enrolment or were not pregnant.

Measures

Maternal sociodemographics and mental health was measured using validated questionnaires administered by trained study staff across two antenatal visits. Mental health assessments included measures of intimate partner violence, depression, childhood trauma, stressful life events and psychological distress. The Intimate Partner Violence (IPV) Questionnaire used in this study was adapted from the WHO multi-country study[32] and the Women's Health Study in Zimbabwe.[33] Participants were dichotomized into exposed or unexposed for having experienced emotional, physical or sexual IPV in the past 12 months; exposure was defined as a score >1 indicating more than an isolated incident within each

For peer review only - http://bmjopen.bmj.com/site/about/guidelines.xhtml

sub-type. The Edinburgh Postnatal Depression Scale (EPDS)[34] was used to measure depression; this scale has been validated for use with pregnant women and in a South African population.[35,36] The EPDS consists of 10 items referring to the past 7 days with each item assessed on a scale from 0 to 3. A total score was obtained by summing responses for all items and was included as a continuous score, with higher scores indicating more severe depressive symptoms; total scores were included in models. To give baseline cohort characteristics depression was dichotomised. A cut of score of ≥13 was used to classify women as depressed.[34] The Childhood Trauma Questionnaire[37] Short-Form was used to assess abuse and neglect experienced as a child. Each item was responded to on a 5 point scale ranging from *1=never true* to *5=very often true*. Continuous scores were used with a total possible range from 28 to 140. Where dichotomised, a cut off score of >36 was used to indicate exposure to childhood trauma, as described in the CTQ manual.[38] The Modified World Mental Health Life Events Questionnaire, adapted based on items used in the South African Stress and Health Study (SASH) in South Africa,[39] to measure stressful or negative life events in the past year (e.g. serious illness, major financial crisis, serious discord with family or friends) Items were scored according to whether or not the event was experienced, *0=no*, *1=yes*. Individual items were then summed to create a total score, ranging from 0 to 17, with higher scores indicating greater exposure to stressful life events. Dichotomous exposure to stressful life events was defined as experiencing at least one such event. The SRQ-20 is a WHO-endorsed measure of psychological distress.[40] The SRQ-20 consists of 20 items, which assess non-psychotic symptoms, including symptoms of depressive and anxiety disorders, scored according to whether or not the symptom was present, *0=no, 1=yes*. Individual items are summed to generate a total score ranging from 0 to 20, with higher scores indicating higher levels of psychological distress.[41] A cut off score of ≥8 was used to classify participants into high versus low risk, as has been used elsewhere.[41,42]

Sociodemographic variables including mother-reported household factors and maternal demographics were collected using an interviewer-administered questionnaire adapted from items used in the SASH Study.[39] Socioeconomic status (SES) was measured based on a composite score of asset ownership, household income, employment and education.[39] Social grants (receiving government support for child care or disability) were self-reported by mothers at enrolment.

Perceived food insecurity was assessed using an adapted version of the U.S. Department of Agriculture (USDA) Short Form Household Food Security Scale[1] which captures food hardship due to financial constraints. Specific questions asked about whether meals were made smaller for children in home, whether children skipped meals or went hungry and whether children in home went a full day without eating - due to limited financial means within the home, as described previously.[28] Questions included referred to children in the home as a conservative estimate of perceived food insecurity; studies have shown that parental buffering often mean that children are the last household members to experience food insecurity.[43,44] Five items were used and an affirmative response to two or more items was coded as being food insecure.

Ethics

Ethical approval was obtained from the Faculty of Health Sciences Research Ethics Committee, University of Cape Town (401/2009) and the Provincial Research committee. Mothers gave written informed consent at enrolment.

Patient and public involvement

For peer review only - http://bmjopen.bmj.com/site/about/guidelines.xhtml

Prior to study initiation, local stakeholders (Department of Health staff and managers) were involved in the planning of the parent study, the Drakenstein Child Health birth cohort study. Patients and public were not involved in conceptualisation or analysis of the specific aims reported in the current study, however, study findings are routinely fedback to the study community.

Statistical Analysis

All data were analysed using SPSS. Univariate logistic regression analyses were conducted to determine the bivariate relationship between food insecurity and demographic and psychosocial predictors. Odds ratios (ORs) with p-values were calculated to determine the strength of these associations. A hierarchical multivariate logistic regression analysis was conducted to independently evaluate IPV exposure and trauma/stress on food insecurity prior to the addition of mental health risk factors, while controlling for demographic variables. Block 1 included community, maternal income, and maternal education. Block 2 included recent experiences of emotional, physical, and sexual IPV as well as maternal childhood trauma and stressful life events. Finally, Block 3 added depression and psychological distress. To determine whether depression played a mediating role on the relationship between IPV and food insecurity, meditational analyses were conducted using PROCESS macro.[45] Model number 4 was used and indirect effects were bootstrapped using 1000 samples. Beta coefficients and standard errors are reported for all paths and 95% confidence intervals are reported for the indirect effects. Models were conducted for the full sample and then for each community individually; models were split by community because of the socioeconomic, cultural, clinical and psychosocial differences between the two communities that could have significant bearings on the results of the mediation models. This process was replicated for depression as a mediator of the relationship between childhood trauma and food insecurity. Mediation models controlled for community, maternal income, maternal education, social grants, number of children in the household and HIV status; childhood trauma was controlled for in all IPV mediation models and emotional, physical, and sexual IPV was controlled for in the childhood trauma mediation models.

Results

A total of 1225 pregnant women were enrolled between March 2012 and February 2015; of these, 992 women had complete data and were included in the analysis. Missing data resulted from non-attendance at the second antenatal visit where psychosocial data was collected. A sensitivity analysis was therefore only done on sociodemographic variables (clinic, education, income, employment, social grants and whether married); those mothers included in the present analysis versus the whole cohort differed significantly only regarding whether mothers received social grants (Supplementary Table 1). Detailed baseline demographic characteristics, stratified by recruitment site, are presented in Table 1. The median age of participants was 26.6 years [standard deviation (SD) 5.8]. The sample was characterized by low SES - 77% of mothers had a monthly income of less than R1,000 (approximately 100 USD), 49% of mothers were receiving social assistance, 26% reported being employed and 38% completed secondary education (high school). A minority of mothers (40%) were married or with a partner. Food security, HIV prevalence and SES quartiles were significantly different between clinics as were the majority of psychosocial variables. Households in Mbekweni were much more likely to be food insecure than households at TC Newman (45.7% versus 12.6%). Mothers at TC Newman were significantly more likely to have experienced emotional and sexual past-year IPV as well as childhood trauma and stressful life events. Co-occurrence of mental health issues was prevalent, though more so at TC Newman. Overall, 12% of mothers had both depression and IPV, 13% depression and childhood trauma and 16% childhood trauma and any form of IPV.

For peer review only - http://bmjopen.bmj.com/site/about/guidelines.xhtml

Table 1. Maternal demographic and psychological variables

	Overall n (%)	TC Newman n (%)	Mbekweni n (%)	X^2	p-value
Number of mothers	992	443	549		
Mean age of mother (SD)	26.6 (5.8)	25.7 (5.4)	27.3 (5.9)	-4.543*	***
Food insecurity					
Secure	685 (69.1)	387 (87.4)	298 (54.3)	125.53	***
Insecure	307 (30.9)	56 (12.6)	251 (45.7)		
Race					
Black	548 (55)	6 (1)	542 (99)	943.05	***
Coloured	443 (45)	437 (99)	6 (1)		
SES Quartiles					
Lowest SES	258 (26)	81 (18)	177 (32)	37.27	***
Low-moderate SES	261 (26)	117 (26)	144 (26)		
Moderate-high SES	242 (24)	109 (25)	133 (24)		
Highest SES	231 (23)	136 (31)	95 (17)		
Maternal Income					
<R1,000/month	767 (77)	330 (74)	437 (80)	7.86	*
R1000-R5000/month	212 (21)	103 (23)	109 (20)		
R5000-R10,000/month	12 (1)	9 (2)	3 (1)		
Receive social assistance	491 (49)	221 (50)	270 (49)	0.085	0.798
Maternal education					
Some secondary	613 (62)	266 (60)	347 (63)	1.037	0.308
Completed Secondary	379 (38)	177 (40)	202 (37)		
Median number of children in household	1	1	1	22.191	**
Married/cohabiting	399 (40)	200 (45)	199 (36)	10.064	*
Employed	254 (26)	132 (30)	122 (22)	7.439	**
Maternal HIV	216 (22)	17 (4)	199 (36)	151.195	***
Psychosocial risk factors					
Past year IPV					
Emotional IPV	266 (27)	155 (35)	111 (20)	27.26	***
Physical IPV	216 (22)	106 (24)	110 (20)	2.18	0.14
Sexual IPV	68 (7)	49 (11)	19 (3)	22.179	***
Probable Depression (EPDS ≥ 13)	242 (24)	112 (25)	130 (24)	0.341	0.559
Childhood trauma	335 (34)	179 (40)	156 (28)	15.761	***
Psychological Distress	208 (21)	109 (25)	99 (18)	6.39	0.011
Stressful Life Events	449 (45)	265 (60)	184 (34)	68.467	***
Co-occurrence of psychosocial risk factors					
Depression & any IPV	122 (12)	68 (15)	54 (10)	6.911	**
Depression & childhood trauma	124 (13)	76 (17)	48 (9)	15.864	***
Childhood trauma & any IPV	154 (16)	101 (23)	53 (10)	32.304	***

*p<0.05, **p<0.01, ***p<0.001; *Note: Psychological risk factors listed where above threshold; IPV above threshold = score of >1 within each subtype (mothers experiencing more than an isolated incidence in past year); Depression above threshold = score ≥ 13; Childhood trauma above threshold where score > 36; Psychological Distress dichotomized into low and high risk categories where high risk = score ≥8; Stressful life events presented where greater than 1.*

For peer review only - http://bmjopen.bmj.com/site/about/guidelines.xhtml

In bivariate analysis (Table 2), antenatal food insecurity was significantly more likely among participants from Mbekweni (aOR 5.82; 95% CI 4.20, 8.07), those who had not completed secondary school (aOR 0.43; 95% CI 0.32, 0.57), mothers with lower income levels (aOR 0.39; 95% CI 0.27, 0.56), mothers who had experienced emotional IPV (aOR 1.44; 95% CI 1.07, 1.94) or physical IPV (aOR 1.84; 95% CI 1.35,

Table 2. Hierarchical logistic regression of variables associated with food insecurity

			Block 1		Block 2		Block 3	
Variables	Unadjusted OR (95% CI)	p-value	Adjusted OR (95% CI)	p-value	Adjusted OR (95% CI)	p-value	Adjusted OR (95% CI)	p-value
Demographic variables								
Community	5.82 (4.20, 8.07)	***	6.02 (4.30, 8.41)	***	8.22 (5.60, 12.06)	***	7.85 (5.29, 11.66)	***
Maternal Income	0.39 (0.27, 0.56)	***	0.42 (0.28, 0.62)	***	0.44 (0.29, 0.66)	***	0.44 (0.29, 0.66)	***
Maternal Education	0.43 (0.32, 0.57)	***	0.42 (0.31, 0.58)	***	0.45 (0.32, 0.63)	***	0.46 (0.33, 0.64)	***
Intimate Partner Violence								
Emotional IPV	1.44 (1.07, 1.94)	*			1.67 (1.09, 2.56)	*	1.60 (1.04, 2.46)	*
Physical IPV	1.84 (1.35, 2.52)	***			1.41 (0.91, 2.18)	0.121	1.32 (0.85, 2.05)	0.216
Sexual IPV	1.62 (0.98, 2.68)	0.061			1.77 (0.92, 3.39)	0.085	1.50 (0.78, 2.89)	0.253
Trauma/Stress+								
Childhood Trauma	1.49 (1.13, 1.97)	**			1.66 (1.18, 2.33)	**	1.52 (1.08, 2.15)	*
Stressful Life Events	0.96 (0.90, 1.02)	0.157			0.98 (0.91, 1.06)	0.585	0.93 (0.86, 1.01)	0.089
Mental Health+								
Depression (EPDS)	1.09 (1.06, 1.12)	***					1.05 (1.01, 1.08)	**
Psychological Distress	1.04 (1.01, 1.08)	*					1.05 (1.00, 1.10)	0.080
			Block X^2(df)	p-value	Block X^2(df)	p-value	Block X^2(df)	p-value
			188.93 (3)	***	8.44 (2)	*	15.75 (2)	***

*p<0.05, **p<0.01, ***p<0.001
+ Trauma/Stress and Mental Health variables were included as continuous scores in regression analyses.

2.52) in the past twelve months and mothers with higher levels of antenatal depression (aOR 1.09; 95% CI 1.06, 1.12), childhood trauma (aOR 1.49; 95% CI 1.13, 1.97) and psychological distress (aOR 1.04; 95% CI 1.01, 1.08).

Hierarchical regression

A hierarchical logistic regression was done to investigate the additive impact of risk factor groups on food security (Table 2). Throughout all blocks, community, maternal education and maternal income remained significantly associated with food insecurity. In block 2 among IPV, trauma and stress risk factors, adjusting for maternal sociodemographic factors and community, emotional IPV and childhood trauma were significant predictors of food insecurity. In the final model (block 3), which incorporated all psychological variables and demographic variables, mothers from Mbekweni were almost eight times (aOR 7.85; 95% CI 5.29, 11.66) as likely as TC Newman mothers to experience antenatal food insecurity. Mothers who completed secondary school were 54% less likely to experience food insecurity (aOR 0.46; 95% CI 0.33, 0.64) compared to mothers who did not complete secondary school. Similarly, mothers with higher incomes were 56% (aOR 0.44; 95% CI 0.29, 0.67) less likely to experience food insecurity.

For peer review only - http://bmjopen.bmj.com/site/about/guidelines.xhtml

For peer review only

Mothers who experienced emotional IPV in the past twelve months were 60% more likely (aOR 1.60; 95% CI 1.04, 2.46), mothers with higher depression scores on EPDS were 5% more likely (aOR 1.05; 95% CI 1.01, 1.08) and mothers with a history of childhood trauma were 52% more likely (aOR 1.52; 95% CI 1.08, 2.15) than mothers without these psychological risk factors to experience food insecurity.

Depression as a mediator of the relationship between IPV and food insecurity

In mediation models including both communities (figure 2a), depression partially mediated the relationship between emotional IPV and food insecurity (direct effect p-value = 0.0001; indirect effect = 0.16, 95% CI 0.07, 0.29) and partially mediated the relationship between physical IPV and food insecurity (direct effect p-value=0.001; indirect effect = 0.17, 95% CI 0.07, 0.28). Sexual IPV was not tested in a mediation model because the bivariate relationship between sexual IPV and food insecurity was not significant (OR 1.62; p=0.06). Mediation models were split by community due to the high significance of recruitment community as an independent predictor of food insecurity; when split by community differing degrees of mediation were found. At TC Newman (figure 2b), depression fully mediated the relationship between emotional IPV and food insecurity (direct effect p-value = 0.13; indirect effect = 0.24, 95% CI 0.05, 0.50) as well as between physical IPV and food insecurity (indirect effect = 0.23, 95% CI 0.06, 0.46). At Mbekweni (figure 2c), depression partially mediated the relationship between emotional IPV and food insecurity (direct effect p-value = 0.0004; indirect effect = 0.11, 95% CI 0.03, 0.25) and partially mediated the relationship between physical IPV and food insecurity (direct effect p-value = 0.008; indirect effect = 0.13, 95% CI 0.04, 0.29).

Depression as a mediator of the relationship between childhood trauma and food insecurity

In mediation models including both communities (figure 3), depression partially mediated the relationship between childhood trauma and food insecurity (direct effect p-value = 0.025; indirect effect = 0.13, 95% CI 0.05, 0.23). These mediation models were also split by community, due to the high significance of community as a risk factor for food insecurity. When applying mediation models to childhood trauma at TC Newman, depression did not mediate the relationship between childhood trauma and food insecurity (direct effect p-value = 0.0009; indirect effect 0.12, 95% CI -0.12, 0.35). Additionally, at Mbekweni, depression did not mediate the relationship between childhood trauma and food insecurity (direct effect p-value = 0.63; indirect effect 0.04, 95% CI -0.02, 0.13).

Discussion

Our goal was to investigate the association between IPV or maternal childhood trauma and food insecurity during pregnancy, as well as to investigate maternal depression as a mediator for these relationships in a LMIC country, South Africa. We found significant effects of emotional IPV and maternal childhood trauma on antenatal food insecurity, after adjusting for community, maternal income and education. Mothers experiencing emotional IPV or with a history of childhood trauma were 60% and 52% more likely, respectively, to live in food insecure households while pregnant. Though previous studies have investigated links between IPV or childhood trauma and food insecurity, the current study extends this research to a low resource setting with a large sample size.

Hernandez and colleagues found that IPV was a significant predictor of food insecurity and that this was mediated by depression.[24] However, this US-based study did not find significant associations between subtypes of IPV and food insecurity - only a composite measure of IPV was found to be significant. Our

For peer review only - http://bmjopen.bmj.com/site/about/guidelines.xhtml

research found that sub-types of IPV were differentially associated with food insecurity, with emotional IPV the only significant predictor in the final model. While mediation models split by site found a mediational effect of depression on this relationship at both clinics; emotional IPV did maintain a direct effect on food insecurity at Mbekweni. This may be an important distinction when planning effective interventions that consider community contexts; qualitative research has found that women feel emotionally abusive acts are more devastating than physical violence.[46] Emotional IPV, therefore, may be a critical and often overlooked risk factor for food insecurity. Further, emotional IPV may manifest differently in a LMIC setting, compared to a high-income setting, where traditional gender norms may affect women's sense of power and identity and therefore, compounded by potential mental health sequelae, may further decrease her ability to manage household resources.[47]

Maternal childhood trauma also emerged as a critical risk factor for food insecurity during pregnancy. Sun and colleagues investigated this link in a large US-based study.[22] These authors reported that childhood trauma was linked to food insecurity during pregnancy, and found that maternal depression modified this relationship. They found a dose-response relationship between number of childhood adverse events and severity of food insecurity; when considered together with depression there was a greater impact on food insecurity. While other high-income country studies have also investigated this association, these have been limited by small sample sizes (n=44, n=31).[48,49] To our knowledge, our study is the first to investigate the link between childhood adversity and food security in a LMIC or mental health as a mediator in this relationship. Childhood trauma measured by family instability, violence exposure at a young age, food insecurity in childhood and neglect is associated with many of the known risk factors of current food insecurity such as lower levels of education, employment and poor mental health outcomes in adulthood.[50] This highlights a critical link between childhood experiences and adult outcomes and the intergenerational effects of trauma. In the context of maternal mental health, this may be particularly relevant as maternal hardship and stress may increase the likelihood of a traumatic childhood for their offspring. As noted by Sun and colleagues, there is an intergenerational transmission of disadvantage, which highlights the need for a multi-faceted approach to address food insecurity.[22] Our findings reveal important intergenerational associations between food insecurity and maternal childhood exposure to violence and suggest that future research is needed to understand how intergenerational transmission of trauma occurs between mothers and children and what can be done to break this cycle. In high-prevalence settings in particular, intervention programs should offer more than nutrition support and should include trauma-informed mental health services to reduce the transmission of trauma from one generation to the next. Though further study is needed to determine if trauma counselling or interventions may help to alleviate the prevalence of food insecurity.

The co-occurrence of psychosocial risk factors was high (12-16%) in our study sample. In order to better understand how these risk factors influence one another, we investigated depression as a mediator in these relationships. In overall models depression partially mediated the relationship between emotional and physical IPV and food insecurity. Notably, the degree to which depression mediated this relationship differed between clinic communities. Depression fully mediated the relationship between emotional and physical IPV and food insecurity at TC Newman, but only partial mediation was found at Mbekweni. This highlights depression as important in the pathway through which IPV affects food insecurity at TC Newman. However, at Mbekweni, though depression also exacerbates this relationship, there may be other factors that explain the significant relationship between IPV and food insecurity. While maternal

For peer review only - http://bmjopen.bmj.com/site/about/guidelines.xhtml

income was controlled for in mediation models, SES quartiles indicate that Mbekweni mothers are economically worse off than TC Newman mothers. This may be impeding the process of food management at Mbekweni, especially in the context of IPV. In overall models investigating depression as a mediator for the relationship between childhood trauma and food security, partial mediation was again seen; however, no mediation was found when models were split by community. At TC Newman, childhood trauma maintained a significant direct effect on food insecurity. It may be that social support networks are more robust at Mbekweni, thus mitigating the downstream effects of childhood trauma in that community. A study in a similar community in South Africa found that social support buffers the effect of trauma on depression symptoms;[51] further, social support has been found to be particularly important for females, compared to males in mitigating mental health outcomes such as depression.[52] Additional research is needed to understand how or why depression mediated the effects of childhood trauma on food insecurity in overall models whereas this effect did not persist when models were split by community.

Additionally, this study indicates that community level factors should be considered when developing nutritional and mental health interventions. Many communities in South Africa are still dealing with the long-term effects of apartheid, this may have a continued effect on stress and mental health in these communities.[53] However, racial disparities exist globally affecting physical and mental health in specific communities differentially to others.[54,55] Specifically, in targeting mental health, contextual factors such as differences in stigma to accessing care, gender norms affecting agency or education levels for women, may have significant differential effects within communities. This community context may be important to understand how to best address key risk factors for food insecurity and to inform design of effective interventions.

Strengths & Limitations

The inclusion criteria for the parent study were broad to ensure generalizability. However, recruitment was done during antenatal care visits, so mothers who did not present for antenatal care or who presented in their third trimester were excluded, which may affect overall generalizability. Further, generalizability may be limited to similar population groups, specifically pregnant mothers and similar communities. In addition, approximately 200 mothers who were enrolled in the study were not included in the analysis due to incomplete data. While it is possible that this subset of mothers is at higher risk for many of the factors investigated; there were not significant differences in key factors investigated for mothers included versus those excluded in the current study. As the current study included cross-sectional data, we cannot assert the direction of causality. While a strength of this study was its investigation of the individual impact of different subtypes of IPV, too few mothers experienced or reported sexual IPV to allow this to be investigated fully. Finally, as all variables used were based on self-report, mothers may have underreported both risk factors and food security due to social desirability bias. Despite these limitations, the current study provides a novel quantitative analysis with a large sample size conducted in a LMIC. Our findings corroborate previous research on risk factors for food insecurity; and build on unpublished data in this cohort by investigating subtypes of IPV as well as maternal depression as a mediator for both childhood trauma and IPV.

Conclusions

Addressing depression during pregnancy through screening and referral services may help to alleviate the negative impact of IPV, childhood trauma and depression on food security; though direction of causality

For peer review only - http://bmjopen.bmj.com/site/about/guidelines.xhtml

cannot be asserted by the current study, significant associations between these variables and food insecurity were found. Both IPV victimization and experiencing childhood trauma were associated with depressive symptoms in mothers, after controlling for maternal income and education. This may impact their household managerial skills by decreasing motivation to obtain food, to find and hold employment and through decreased physical and cognitive functioning. Our findings highlight the importance of comprehensive programs aimed at nutrition support or food security, as well as the importance of addressing multiple concurrent psychosocial risk factors which may help to reduce food insecurity and alleviate its negative impact on child health. However, notably, mediation models indicate that the effects of maternal mental health issues are experienced differently at the two communities in this cohort highlighting the importance of program and policy efforts targeted to specific community profiles. Notably, though not the focus of the current study, both maternal income and maternal education were highly correlated with food insecurity – education especially is likely an important factor cross cutting all key risk and outcome variables – promoting educational opportunities represents an important intervention to improve maternal and thus child health.

Acknowledgements
We thank the study staff in Paarl, the study data team and lab teams, the clinical and administrative staff of the Western Cape Government Health Department at Paarl Hospital and at the clinics for support of the study. We acknowledge the advice from members of the study International Advisory Board and thank our collaborators. We thank the families and children who participated in this study.

Declaration of interest
The authors have no conflicts of interest to declare.

Funding
The study was funded by the Bill and Melinda Gates Foundation [OPP 1017641] with support from the National Institutes of Health (H3Africa 1U01AI110466-01A1). Additional support for DJS, NK, WB and HJZ, and for research reported in this publication was from the South African Medical Research Council (SAMRC) and the UK Royal Society, Newton Advanced Fellowship. WB is supported by the SAMRC National Health Scholars programme; NK receives support from the SAMRC under a Self-Initiated Research Grant. The views and opinions expressed are those of the authors and do not necessarily represent the official views of the SAMRC.

Author contributions:
WB & JP conceptualized the analysis and wrote the first draft of the manuscript. HJZ is principal investigator of the parent study; DS leads the psychosocial study aspects; NK and KD are co-investigators and contributed to the study design and implementation. JP conducted the data analysis. CK, JP and DS provided critical inputs on the manuscript. All authors read and approved the final manuscript.

Data sharing statement: No additional data are available. All study instruments used in this analysis are available to requesting researchers.

For peer review only

References

1. Bickel G, Nord M, Price C, Hamilton W, Cook J, Guide to Measuring Household Food Security, Revised 2000. U.S. Department of Agriculture, Food and Nutrition Service, Alexandria VA. March, 2000.
2. FAO, IFAD, UNICEF, WFP and WHO. 2017. The State of Food Security and Nutrition in the World 2017. Building resilience for peace and food security. Rome, FAO.
3. Borders AE, Grobman WA, Amsden LB, Holl JL. Chronic stress and low birth weight neonates in a low-income population of women. *Obstet gynecol* 2007; **109**(2 Pt 1): 331-8.
4. Laraia BA, Siega-Riz AM, Gundersen C. Household food insecurity is associated with self-reported pregravid weight status, gestational weight gain, and pregnancy complications. *J Am Diet Assoc* 2010; **110**(5): 692-701.
5. Cook JT, Black M, Chilton M, et al. Are food insecurity's health impacts underestimated in the U.S. population? Marginal food security also predicts adverse health outcomes in young U.S. children and mothers. *Advances in nutrition (Bethesda, Md)* 2013; **4**(1): 51-61.
6. Cook JT, Frank DA, Levenson SM, et al. Child food insecurity increases risks posed by household food insecurity to young children's health. *J Nutr* 2006; **136**(4): 1073-6.
7. Bronte-Tinkew J, Zaslow M, Capps R, Horowitz A, McNamara M. Food insecurity works through depression, parenting, and infant feeding to influence overweight and health in toddlers. *J Nutr* 2007; **137**(9): 2160-5.
8. Cook JT, Frank DA, Berkowitz C, et al. Food insecurity is associated with adverse health outcomes among human infants and toddlers. *J Nutr* 2004; **134**(6): 1432-8.
9. Zaslow M, Bronte-Tinkew J, Capps R, Horowitz A, Moore KA, Weinstein D. Food security during infancy: implications for attachment and mental proficiency in toddlerhood. *Matern Child Health J* 2009; **13**(1): 66-80.
10. Hernandez DC, Jacknowitz A. Transient, but not persistent, adult food insecurity influences toddler development. *J Nutr* 2009; **139**(8): 1517-24.
11. Rose-Jacobs R, Black MM, Casey PH, et al. Household food insecurity: associations with at-risk infant and toddler development. *Pediatrics* 2008; **121**(1): 65-72.
12. Kirkpatrick SI, McIntyre L, Potestio ML. Child hunger and long-term adverse consequences for health. *Arch Pediatr Adoles Med* 2010; **164**(8): 754-62.
13. Ivers LC, Cullen KA. Food insecurity: special considerations for women. *Am J Clin Nutr* 2011; **94**(6): 1740S-4S.
14. Davison KM, Kaplan BJ. Food insecurity in adults with mood disorders: prevalence estimates and associations with nutritional and psychological health. *Ann Gen Psychiatry* 2015; **14**: 21.
15. Martin MS, Maddocks E, Chen Y, Gilman SE, Colman I. Food insecurity and mental illness: disproportionate impacts in the context of perceived stress and social isolation. *Pub Health* 2016.
16. Melchior M, Caspi A, Howard LM, et al. Mental health context of food insecurity: a representative cohort of families with young children. *Pediatrics* 2009; **124**(4): e564-72.
17. Sorsdahl K, Slopen N, Siefert K, Seedat S, Stein DJ, Williams DR. Household food insufficiency and mental health in South Africa. *J Epidem Community Health* 2011; **65**(5): 426-31.
18. Koen N, Wyatt GE, Williams JK, et al. Intimate partner violence: associations with low infant birthweight in a South African birth cohort. *Metab Brain Dis* 2014; **29**(2): 281-99.
19. Brittain K, Myer L, Koen N, et al. Risk Factors for Antenatal Depression and Associations with Infant Birth Outcomes: Results From a South African Birth Cohort Study. *Paediatr Perinat Epidemiol* 2015.
20. Ross J, Hanlon C, Medhin G, et al. Perinatal mental distress and infant morbidity in Ethiopia: a cohort study. *Archives of Disease in Childhood: Fetal and Neonatal Edition* 2011; **96**(1): F59-64.
21. Rahman A, Harrington R, Bunn J. Can maternal depression increase infant risk of illness and growth impairment in developing countries? *Child Care Health Dev* 2002; **28**(1): 51-6.

For peer review only

22. Sun J, Knowles M, Patel F, Frank DA, Heeren TC, Chilton M. Childhood Adversity and Adult Reports of Food Insecurity Among Households With Children. *Am J Prev Med* 2015.
23. Chilton M, Knowles M, Rabinowich J, Arnold KT. The relationship between childhood adversity and food insecurity: 'It's like a bird nesting in your head'. *Public Health Nutr* 2015; **18**(14): 2643-53.
24. Hernandez DC, Marshall A, Mineo C. Maternal depression mediates the association between intimate partner violence and food insecurity. *J Women's Health* 2014; **23**(1): 29-37.
25. Gibbs A, Corboz J, Jewkes R. Factors associated with recent intimate partner violence experience amongst currently married women in Afghanistan and health impacts of IPV: a cross sectional study. *BMC Pub Health* 2018; **18**.
26. Wehler C, Weinreb LF, Huntington N, et al. Risk and protective factors for adult and child hunger among low-income housed and homeless female-headed families. *Am J Public Health* 2004; **94**(1): 109-15.
27. Radimer KL, Olson CM, Greene JC, Campbell CC, Habicht J-P. Understanding hunger and developing indicators to assess it in women and children. *J Nutr Educ* 1992; **24**(1): 36S-44S.
28. Pellowski JA, Barnett W, Kuo CC, Koen N, Zar HJ, Stein DJ. Investigating tangible and mental resources as predictors of perceived household food insecurity during pregnancy among women in a South African birth cohort study. *Soc Sci Med* 2017; **187**: 76-84.
29. Statistics South Africa. (Sept 2018). *Drakenstein Municipality*. Retrieved from http://www.statssa.gov.za/?page_id=993&id=drakenstein-municipality.
30. Zar HJ, Barnett W, Myer L, Stein DJ, Nicol MP. Investigating the early-life determinants of illness in Africa: the Drakenstein Child Health Study. *Thorax* 2015; **70**(6): 592-4.
31. Stein DJ, Koen N, Donald KA, et al. Investigating the psychosocial determinants of child health in Africa: The Drakenstein Child Health Study. *J Neurosci Meth* 2015.
32. Jewkes R. Intimate partner violence: causes and prevention. *Lancet (London, England)* 2002; **359**(9315): 1423-9.
33. Shamu S, Abrahams N, Temmerman M, Musekiwa A, Zarowsky C. A systematic review of African studies on intimate partner violence against pregnant women: prevalence and risk factors. *PloS one* 2011; **6**(3): e17591.
34. Cox JL, Holden JM, Sagovsky R. Detection of postnatal depression. Development of the 10-item Edinburgh Postnatal Depression Scale. *Br J Psychiatry* 1987; **150**: 782-6.
35. Lawrie TA, Hofmeyr GJ, de Jager M, Berk M. Validation of the Edinburgh Postnatal Depression Scale on a cohort of South African women. *S Afr Med J* 1998; **88**(10): 1340-4.
36. Tsai AC, Scott JA, Hung KJ, et al. Reliability and validity of instruments for assessing perinatal depression in African settings: systematic review and meta-analysis. *PLoS One* 2013; **8**(12): e82521.
37. Bernstein DP, Fink L, Handelsman L, et al. Initial reliability and validity of a new retrospective measure of child abuse and neglect. *Am J Psych* 1994; **151**(8): 1132-6.
38. Bernstein D, Fink L. Childhood Trauma Questionnaire: a retrospective self-report. San Antonio: The Psychological Corporation; 1998.
39. Myer L, Stein DJ, Grimsrud A, Seedat S, Williams DR. Social determinants of psychological distress in a nationally-representative sample of South African adults. *Soc Sci Med (1982)* 2008; **66**(8): 1828-40.
40. Beusenberg M, Orley J. A user's guide to the self reporting questionnaire (SRQ). Division of Mental Health. Geneva: World Health Organization: 1994.
41. Ventevogel P, De Vries G, Scholte WF, et al. Properties of the Hopkins Symptom Checklist-25 (HSCL-25) and the Self-Reporting Questionnaire (SRQ-20) as screening instruments used in primary care in Afghanistan. *J Soc Psychiatry* 2007; **42**(4): 328-35.
42. Harpham T, Reichenheim M, Oser R, et al. Measuring mental health in a cost-effective manner. *Health Policy Plan* 2003; **18**(3): 344-9.

For peer review only

43. Hadley C, Lindstrom D, Tessema F, Belachew T. Gender Bias in the Food Insecurity Experience of Ethiopian Adolescents. *Soc Sci Med (1982)* 2008; **66**(2): 427-38.
44. Leonard WR. Household-level strategies for protecting children from seasonal food scarcity. *Soc Sci Med (1982)* 1991; **33**(10): 1127-33.
45. Hayes AF. Introduction to Mediation, Moderation, and Conditional Process Analysis: A Regression-Based Approach. *Guilford Press* 2013.
46. World Health Organization. WHO multi-country study on women's health and domestic violence against women: summary report of initial results on prevalence, health outcomes and women's responses. Geneva, WHO, 2012.
47. Department of Women, Children & People with Disabilities (DWCPD). Stop violence against women in South Africa. Cape Town, DWCPD, 2013.
48. Chilton MM, Rabinowich JR, Woolf NH. Very low food security in the USA is linked with exposure to violence. *Public Health Nutr* 2014; **17**(1): 73-82.
49. Chilton M, Knowles M, Bloom SL. The Intergenerational Circumstances of Household Food Insecurity and Adversity. *J Hunger Environ Nutr* 2017; **12**(2): 269-97.
50. Messina N, Grella C. Childhood trauma and women's health outcomes in a California prison population. *Am J Pub Health* 2006; **96**(10): 1842-8.
51. Casale M, Wild L, Cluver L, Kuo C. Social support as a protective factor for depression among women caring for children in HIV-endemic South Africa. *J Behav Med* 2015; **38**(1): 17-27.
52. Kendler KS, Myers J, Prescott CA. Sex differences in the relationship between social support and risk for major depression: a longitudinal study of opposite-sex twin pairs. *Am J Psychiatry* 2005; **162**(2): 250-6.
53. Jackson PB, Williams DR, Stein DJ, Herman A, Williams SL, Redmond DL. Race and Psychological Distress: The South African Stress and Health Study. *J Health Soc Behav* 2010; **51**(4): 458-77.
54. Penner LA, Hagiwara N, Eggly S, Gaertner SL, Albrecht TL, Dovidio JF. Racial Healthcare Disparities: A Social Psychological Analysis. *Eur Rev Soc Psych* 2013; **24**(1): 70-122.
55. Flores G, Lin H. Trends in racial/ethnic disparities in medical and oral health, access to care, and use of services in US children: has anything changed over the years? *Int J Equity Health* 2013; **12**: 10.

Figure legends

Fig 2a-2c. Covariates included in the model are: community, maternal income, maternal education, social grants, number of children in the household, HIV status and childhood trauma.

Fig 3. Mediation models for both communities, for TC Newman and for Mbekweni investigating depression as a mediator for childhood trauma and food insecurity. Depression **partially** mediated the overall relationship between childhood trauma and food insecurity, but **did not mediate** the relationship at TC Newman and **did not** mediate the relationship at Mbekweni. Covariates included in the model are: community, maternal income, maternal education, social grants, number of children in the household, HIV status and emotional, physical and sexual forms of IPV.

For peer review only

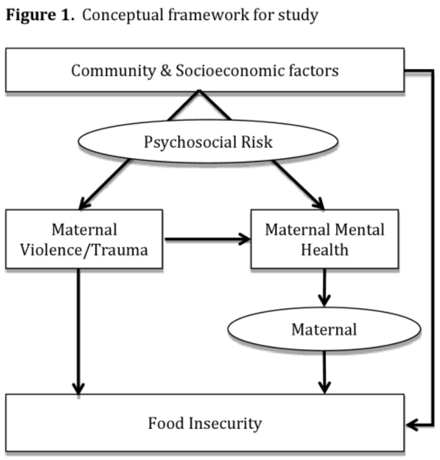

Figure 1. Conceptual framework for study.

209x297mm (300 x 300 DPI)

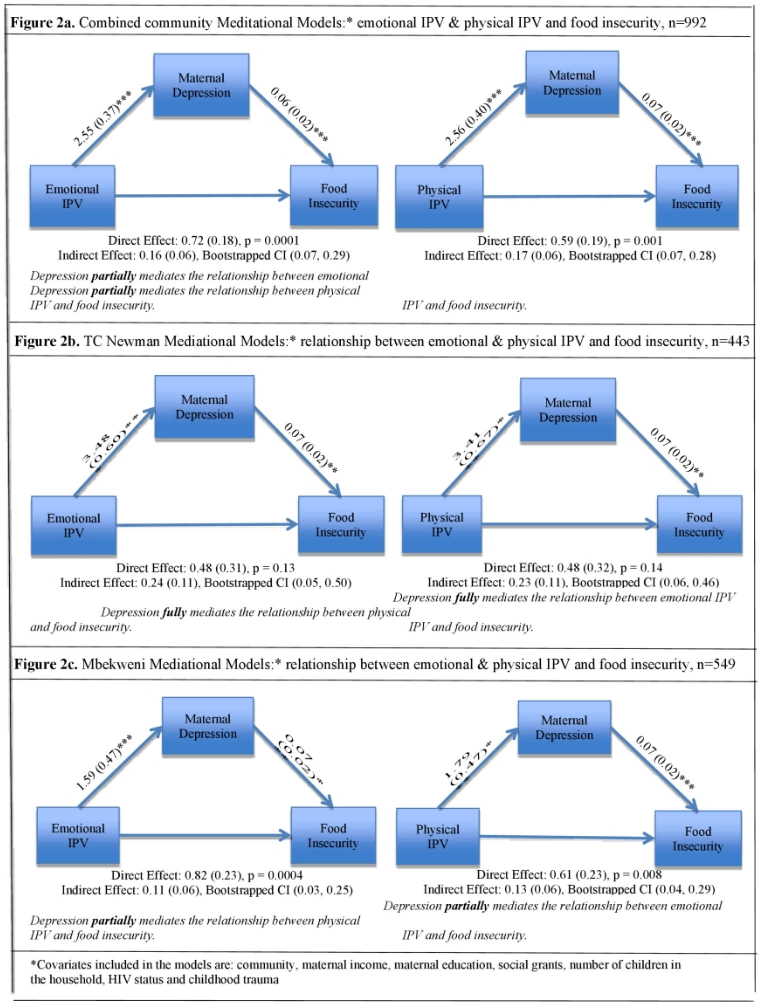

Fig 2a-2c. Covariates included in the model are: community, maternal income, maternal education, social grants, number of children in the household, HIV status and childhood trauma.

209x297mm (300 x 300 DPI)

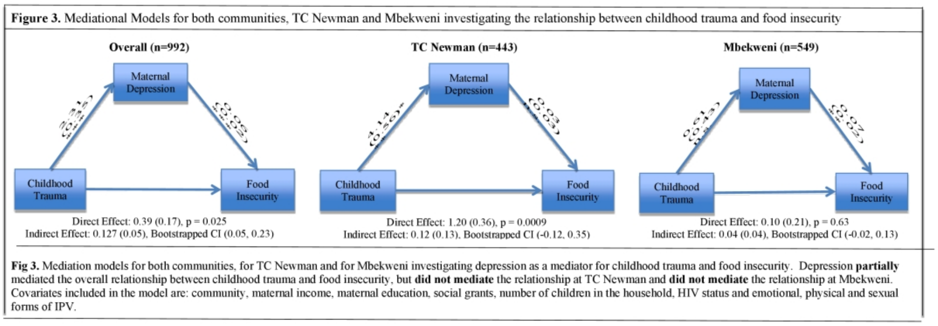

Fig 3. Mediation models for both communities, for TC Newman and for Mbekweni investigating depression as a mediator for childhood trauma and food insecurity. Depression partially mediated the overall relationship between childhood trauma and food insecurity, but did not mediate the relationship at TC Newman and did not mediate the relationship at Mbekweni. Covariates included in the model are: community, maternal income, maternal education, social grants, number of children in the household, HIV status and emotional, physical and sexual forms of IPV.

297x209mm (300 x 300 DPI)

Supplemental Table 1. Sensitivity analysis: comparison of key sociodemographic variables between participants included in and excluded from the current study.

	Included sample	Excluded sample	p-value
Number of mothers	992	233	
Clinic			
Mbekweni	549 (55)	131 (56)	
TC Newman	443 (45)	102 (44)	0.808
Race			
Black	548 (55)	132 (57)	
Coloured	443 (45)	101 (43)	0.708
Maternal Income			
<R1,000/month	767 (77)	168 (78)	
R1000-R5000/month	212 (21)	44 (20)	0.954
R5000-R10,000/month	12 (1)	3 (1)	
Receive social assistance	491 (49)	92 (40)	0.005
Maternal education			
Some secondary	613 (62)	127 (55)	
Completed Secondary	379 (38)	103 (45)	0.066
Median number of children in household	1	1	0.911
Married/cohabiting	399 (40)	92 (40)	0.828
Employed	254 (26)	67 (29)	0.429

For peer review only

STROBE Statement

Checklist of items that should be included in reports of observational studies

Section/Topic	Item No	Recommendation	Reported on Page No
**Title and abstract**	1	(*a*) Indicate the study's design with a commonly used term in the title or the abstract	2
		(*b*) Provide in the abstract an informative and balanced summary of what was done and what was found	2
**Introduction**			
Background/rationale	2	Explain the scientific background and rationale for the investigation being reported	3
Objectives	3	State specific objectives, including any prespecified hypotheses	3
**Methods**			
Study design	4	Present key elements of study design early in the paper	4
Setting	5	Describe the setting, locations, and relevant dates, including periods of recruitment, exposure, follow-up, and data collection	4
Participants	6	*Cross-sectional study*—Give the eligibility criteria, and the sources and methods of selection of participants	4,5
Variables	7	Clearly define all outcomes, exposures, predictors, potential confounders, and effect modifiers. Give diagnostic criteria, if applicable	4,5
Data sources/measurement	8*	For each variable of interest, give sources of data and details of methods of assessment (measurement). Describe comparability of assessment methods if there is more than one group	4,5
Bias	9	Describe any efforts to address potential sources of bias	10
Study size	10	Explain how the study size was arrived at	5
Quantitative variables	11	Explain how quantitative variables were handled in the analyses. If applicable, describe which groupings were chosen and why	4,5
Statistical methods	12	(*a*) Describe all statistical methods, including those used to control for confounding	5
		(*b*) Describe any methods used to examine subgroups and interactions	5,7
		(*c*) Explain how missing data were addressed	5
		(*d*) *Cohort study*—If applicable, explain how loss to follow-up was addressed<br>*Case-control study*—If applicable, explain how matching of cases and controls was addressed<br>*Cross-sectional study*—If applicable, describe analytical methods taking account of sampling strategy	n/a
		(*e*) Describe any sensitivity analyses	10

For peer review only - http://bmjopen.bmj.com/site/about/guidelines.xhtml

Section/Topic	Item No	Recommendation	Reported on Page No
**Results**			
Participants	13*	(a) Report numbers of individuals at each stage of study—eg numbers potentially eligible, examined for eligibility, confirmed eligible, included in the study, completing follow-up, and analysed	5
		(b) Give reasons for non-participation at each stage	
		(c) Consider use of a flow diagram	n/a
Descriptive data	14*	(a) Give characteristics of study participants (eg demographic, clinical, social) and information on exposures and potential confounders	6
		(b) Indicate number of participants with missing data for each variable of interest	5
		(c) *Cohort study*—Summarise follow-up time (eg, average and total amount)	n/a
Outcome data	15*	*Cohort study*—Report numbers of outcome events or summary measures over time	n/a
		Case-control study—Report numbers in each exposure category, or summary measures of exposure	
		Cross-sectional study—Report numbers of outcome events or summary measures	6
Main results	16	(*a*) Give unadjusted estimates and, if applicable, confounder-adjusted estimates and their precision (eg, 95% confidence interval). Make clear which confounders were adjusted for and why they were included	7
		(*b*) Report category boundaries when continuous variables were categorized	6
		(*c*) If relevant, consider translating estimates of relative risk into absolute risk for a meaningful time period	n/a
Other analyses	17	Report other analyses done—eg analyses of subgroups and interactions, and sensitivity analyses	8,10
**Discussion**			
Key results	18	Summarise key results with reference to study objectives	8
Limitations	19	Discuss limitations of the study, taking into account sources of potential bias or imprecision. Discuss both direction and magnitude of any potential bias	10
Interpretation	20	Give a cautious overall interpretation of results considering objectives, limitations, multiplicity of analyses, results from similar studies, and other relevant evidence	8,9,10
Generalisability	21	Discuss the generalisability (external validity) of the study results	10
**Other Information**			
Funding	22	Give the source of funding and the role of the funders for the present study and, if applicable, for the original study on which the present article is based	10

**Give information separately for cases and controls in case-control studies and, if applicable, for exposed and unexposed groups in cohort and cross-sectional studies.*

Note: An Explanation and Elaboration article discusses each checklist item and gives methodological background and published examples of transparent reporting. The STROBE checklist is best used in conjunction with this article (freely available on the Web sites of PLoS Medicine at http://www.plosmedicine.org/, Annals of Internal Medicine at http://www.annals.org/, and Epidemiology at http://www.epidem.com/). Information on the STROBE Initiative is available at www.strobe-statement.org.

For peer review only - http://bmjopen.bmj.com/site/about/guidelines.xhtml

Food insecure pregnant women in South Africa: A cross-sectional exploration of maternal depression as a mediator of violence and trauma risk factors.

Journal:	*BMJ Open*
Manuscript ID	bmjopen-2017-018277.R2
Article Type:	Research
Date Submitted by the Author:	17-Jan-2019
Complete List of Authors:	Barnett, Whitney; University of Cape Town, Department of Paediatrics and Child Health, Red Cross War Memorial Children's Hospital; South African Medical Research Council Unit on Child & Adolescent Health<br>Pellowski, Jennifer; Brown University School of Public Health, Department of Behavioral and Social Sciences and International Health Institute<br>Kuo, Caroline; Brown University, Department of Behavioral and Social Sciences and Center for Alcohol and Addiction Studies<br>Koen, Nastassja; University of Cape Town, Department of Psychiatry and Mental Health; South African Medical Research Council Unit on Risk and Resilience in Mental Disorders<br>Donald, Kirsten; University of Cape Town, Division of Developmental Paediatrics, Department of Paediatrics & Child Health, Red Cross War Memorial Children's Hospital<br>Zar, Heather; University of Cape Town, Department of Paediatrics and Child Health, Red Cross War Memorial Children's Hospital; South African Medical Research Council, Unit on Child and Adolescent Health<br>Stein, Dan; University of Cape Town, Department of Psychiatry and Mental Health; South African Medical Research Council Unit on Risk and Resilience in Mental Disorders
**Primary Subject Heading**:	Public health
Secondary Subject Heading:	Nutrition and metabolism, Mental health
Keywords:	food insecurity, maternal mental health, intimate partner violence, depression mediation

Food insecure pregnant women in South Africa: A cross-sectional exploration of maternal depression as a mediator of violence and trauma risk factors.

Authors: Whitney Barnett[1], Jennifer Pellowski[2], Caroline Kuo[3,4], Nastassja Koen[4,5], Kirsten A Donald[6], Heather J. Zar[1], Dan J. Stein[4,5]

[1] Department of Paediatrics and Child Health, Red Cross War Memorial Children's Hospital, and Medical Research Council Unit on Child & Adolescent Health, University of Cape Town, South Africa,
[2] Department of Behavioral and Social Sciences and International Health Institute, Brown University School of Public Health, Providence, Rhode Island, USA [3]Department of Behavioral and Social Sciences and Center for Alcohol and Addiction Studies, Brown University, Providence Rhode Island, USA [4]Department of Psychiatry and Mental Health, University of Cape Town, South Africa [5]South African Medical Research Council Unit on Risk and Resilience in Mental Disorders, Cape Town, South Africa [6]Division of Developmental Paediatrics, Department of Paediatrics & Child Health, Red Cross War Memorial Children's Hospital, University of Cape Town, Cape Town, South Africa

Corresponding author:
Whitney Barnett
Department of Paediatrics and Child Health
Unit B11, REACH Center
Red Cross War Memorial Children's Hospital
Cape Town, South Africa
barnett.whitney@gmail.com
Tel: +27 (0) 790432604

Key words: food insecurity, maternal mental health, intimate partner violence, depression, mediation

Word count: 4,462

For peer review only

Abstract

Objectives: Better understanding of psychosocial risk factors for food insecurity (FI) during pregnancy, and how they interact is crucial, given long-term health implications for maternal and child health. We investigated the association between maternal childhood trauma as well as intimate partner violence (IPV) and FI among pregnant women in South Africa, in the Drakenstein Child Health Study, and whether maternal depression mediates these relationships.

Setting: Two primary care clinics in Paarl, South Africa.

Participants: 992 pregnant women; inclusion criteria were clinic attendance and remaining in area for at least 1 year; women were excluded if a minor.

Methods: We examined psychosocial predictors of FI using multivariate regression. Mediation analyses investigated whether depression mediated the relationship between IPV and FI as well as between childhood trauma and FI, including disaggregation by two study communities. FI was assessed using an adapted US Department of Agriculture food security scale; households were coded as food insecure where 2 of 5 affirmative responses were recorded.

Results: Among 992 pregnant women there were high rates of IPV (7-27%), depression (24%), and childhood trauma (34%). In multivariate cross-sectional analysis, emotional IPV (aOR 1.60; 95% CI 1.04,2.46), depression (aOR 1.05; 95% CI 1.01,1.08) and childhood trauma (aOR 1.52; 95% CI 1.08,2.15) predicted FI. In mediation models depression partially mediated the relationship between emotional IPV and FI as well as physical IPV and FI; depression partially mediated the relationship between childhood trauma and FI. Differing degrees of mediation were found when applied to communities.

Conclusions: Antenatal maternal depression, IPV and childhood trauma were highly prevalent and associated with FI. Depression, IPV and trauma screening services should be considered within routine antenatal care and may offer an opportunity to identify and intervene. Community level differences in risk and in mediation analyses indicate that contextual tailoring of interventions may be important.

Strengths & Limitations of this study:

- There are few studies investigating depression as a mediator in relationships between subtypes of maternal intimate partner violence or childhood trauma and food insecurity during pregnancy.
- This study extends existing related research to a low resource African population with a large sample size.
- The current study was a cross-sectional analysis; therefore further research is needed to assess the direction of causality, and if differences exist by trimester and postpartum.

For peer review only - http://bmjopen.bmj.com/site/about/guidelines.xhtml

For peer review only

Background

Food insecurity is the lack of nutritionally adequate and safe food or a limited ability to acquire necessary food in socially acceptable ways.[1] The Food and Agriculture Organization (FAO) estimates that 689 million people worldwide (1 in 10) suffer from severe food insecurity (2014-2016); Africa has the highest prevalence of severe food insecurity (27.4%), almost four times the prevalence of other regions.[2] Studies have shown a link between food insecurity and poor pregnancy outcomes, including low birth weight,[3] gestational diabetes, and pregnancy complications.[4] In addition, young children in food insecure households have poorer general health[5-7] increased probability of being hospitalized,[6,8] lower levels of parent-child attachment,[9] and increased developmental delays.[9-11] Chronic hunger in childhood has also been linked to a higher likelihood of chronic medical conditions, such asthma, heart conditions, kidney disease or allergies.[12] Pregnant women may be particularly vulnerable to food insecurity due to increased nutrient demands and the inability to continue working, leading to financial strain.

Maternal mental health disorders are prevalent in low and middle-income countries (LMICs). Maternal mental health problems such as depression[13] and psychosocial risk factors such as stressful life events, intimate partner violence and trauma[14-17] are associated with food insecurity as well as poorer pregnancy outcomes such as low infant birth weight,[18] impaired foetal[19] and infant growth and nutritional status[3,20] as well as poorer infant cognitive development.[8,21] Although the relationship between maternal trauma or violence exposures as well as mental health and food insecurity has been explored, few studies have investigated depression as a mediator in the relationship between other psychosocial risk factors (e.g. violence or trauma) and food insecurity. Sun and colleagues, in a large US based study, found maternal childhood trauma to be linked to food insecurity during pregnancy and that depression mediated this relationship.[22] Others in the US have found similar links between childhood trauma and food insecurity, but have not investigated mental health pathways.[23] In another US based study, IPV was found to be a significant predictor of food insecurity, mediated by depression.[24] However, this study did not find differential associations between sub-types of IPV (emotional, physical and sexual), though others have.[25] The majority of studies have focused on high-income countries[14,16,22,24,26] or have used small sample sizes to explore associations.[14,23] The current study aims to extend previous research to a LMIC context and to analyse multiple exposures, maternal trauma, IPV and stressful events, which are often co-occuring and have a higher prevalence in LMIC settings in a large study sample.

Examining maternal psychosocial risk factors and mental health characteristics in relation to food insecurity in LMICs is important. Particularly in the context of high proportions of maternal headed and single parent households and given the high prevalence of maternal psychosocial risk factors, especially during pregnancy, when exposures can adversely affect both maternal and child long-term health. Food security is a managed process such that family members have some control over how they cope with food insecurity and who within the family experiences it.[26,27] This ability to manage the effects of food insecurity may be adversely affected by maternal psychosocial risk factors and maternal mental health.[16] Further, community level factors such as differences in stigma to accessing care, gender norms affecting agency or education levels for women, may have significant differential effects within communities. This community context may be important to understand how to best address key risk factors for food insecurity and to inform design of effective interventions.

For peer review only - http://bmjopen.bmj.com/site/about/guidelines.xhtml

We therefore aim to explore associations between maternal psychosocial risk factors or mental health and food insecurity, disaggregated by two communities with different risk profiles and community level factors. We investigate whether depression acts as a mediator in the relationship between IPV or childhood trauma and food insecurity in a LMIC context,(see figure 1: conceptual framework). This extends the evidence base to geographic regions where these issues are highly prevalent but the relationships between these variables are poorly understood and rarely investigated.

Given the long-term health implications of food insecurity for child development as well as maternal and child mental and physical health,[12-15] understanding how risk factors for poor child health outcomes interact is critical to inform public policy to address the most urgent modifiable risk factors. Previous published findings from this cohort have shown links between psychosocial risk factors and food insecurity during pregnancy;[28] this paper builds upon that research by investigating the mediational effects of maternal depression on the relationship between emotional, physical and sexual IPV and food insecurity as well as maternal childhood trauma and food insecurity. Addressing food insecurity during pregnancy offers an opportunity to link antenatal care with nutritional programs and manage associated mental health risk factors at a time when those risk factors impact not only the mother's safety and well being but also infant outcomes after birth.

Methods

The Drakenstein Child Health Study (DCHS) is a multidisciplinary population-based birth cohort study located in a peri-urban area, 60 km outside of Cape Town, South Africa. It is a low socioeconomic community comprising approximately 200 000 people predominantly of mixed-ancestry (62.5%; 13.5% caucasian; 22.7% Black African).[29] The district is characterized by a high prevalence of a range of health risk factors such as single-parent households, depression, childhood trauma, IPV, poverty, low levels of education (27.4% completing secondary school) and high unemployment (17.6%). The DCHS is a longitudinal cohort study following mother-child dyads through early childhood.[30,31] The current analysis utilises data from two antenatal visits: maternal psychosocial health and food security were measured at an antenatal visit between 28 to 32 weeks' gestation; sociodemographics were measured at the enrolment visit, at 20-28 weeks' gestation.

Participants

Pregnant women were enrolled from March 2012 to March 2015. Women were enrolled in their second trimester, between 20 to 28 weeks' gestation at two public sector primary health care clinics, one serving a predominantly mixed-ancestry population (TC Newman) and the other serving a predominantly Black African population (Mbekweni). Inclusion criteria were 1) attendance at one of the two study clinics 2) and 2) intending to remain in the study area for at least 1 year. Mothers were excluded if they were under 18 years of age at enrolment or were not pregnant.

Measures

Maternal sociodemographics and mental health was measured using validated questionnaires administered by trained study staff across two antenatal visits. Mental health assessments included measures of intimate partner violence, depression, childhood trauma, stressful life events and psychological distress. The Intimate Partner Violence (IPV) Questionnaire used in this study was adapted from the WHO multi-country study[32] and the Women's Health Study in Zimbabwe.[33] Participants were dichotomized into exposed or unexposed for having experienced emotional, physical or sexual IPV in the

For peer review only - http://bmjopen.bmj.com/site/about/guidelines.xhtml

past 12 months; exposure was defined as a score >1 indicating more than an isolated incident within each sub-type. The Edinburgh Postnatal Depression Scale (EPDS)[34] was used to measure depression; this scale has been validated for use with pregnant women and in a South African population.[35,36] The EPDS consists of 10 items referring to the past 7 days with each item assessed on a scale from 0 to 3. A total score was obtained by summing responses for all items and was included as a continuous score, with higher scores indicating more severe depressive symptoms; total scores were included in models. To give baseline cohort characteristics depression was dichotomised. A cut of score of ≥13 was used to classify women as depressed.[34] The Childhood Trauma Questionnaire[37] Short-Form was used to assess abuse and neglect experienced as a child. Each item was responded to on a 5 point scale ranging from *1=never true* to *5=very often true*. Continuous scores were used with a total possible range from 28 to 140. Where dichotomised, a cut off score of >36 was used to indicate exposure to childhood trauma, as described in the CTQ manual.[38] The Modified World Mental Health Life Events Questionnaire, adapted based on items used in the South African Stress and Health Study (SASH) in South Africa,[39] to measure stressful or negative life events in the past year (e.g. serious illness, major financial crisis, serious discord with family or friends) Items were scored according to whether or not the event was experienced, *0=no*, *1=yes*. Individual items were then summed to create a total score, ranging from 0 to 17, with higher scores indicating greater exposure to stressful life events. Dichotomous exposure to stressful life events was defined as experiencing at least one such event. The SRQ-20 is a WHO-endorsed measure of psychological distress.[40] The SRQ-20 consists of 20 items, which assess non-psychotic symptoms, including symptoms of depressive and anxiety disorders, scored according to whether or not the symptom was present, *0=no, 1=yes*. Individual items are summed to generate a total score ranging from 0 to 20, with higher scores indicating higher levels of psychological distress.[41] A cut off score of ≥8 was used to classify participants into high versus low risk, as has been used elsewhere.[41,42]

Sociodemographic variables including mother-reported household factors and maternal demographics were collected using an interviewer-administered questionnaire adapted from items used in the SASH Study.[39] Socioeconomic status (SES) was measured based on a composite score of asset ownership, household income, employment and education.[39] Social grants (receiving government support for child care or disability) were self-reported by mothers at enrolment.

Perceived food insecurity was assessed using an adapted version of the U.S. Department of Agriculture (USDA) Short Form Household Food Security Scale[1] which captures food hardship due to financial constraints. Specific questions asked about whether meals were made smaller for children in home, whether children skipped meals or went hungry and whether children in home went a full day without eating - due to limited financial means within the home, as described previously.[28] Questions included referred to children in the home as a conservative estimate of perceived food insecurity; studies have shown that parental buffering often mean that children are the last household members to experience food insecurity.[43,44] Five items were used and an affirmative response to two or more items was coded as being food insecure.

Ethics

Ethical approval was obtained from the Faculty of Health Sciences Research Ethics Committee, University of Cape Town (401/2009) and the Provincial Research committee. Mothers gave written informed consent at enrolment.

For peer review only - http://bmjopen.bmj.com/site/about/guidelines.xhtml

Patient and public involvement

Prior to study initiation, local stakeholders (Department of Health staff and managers) were involved in the planning of the parent study, the Drakenstein Child Health birth cohort study. Patients and public were not involved in conceptualisation or analysis of the specific aims reported in the current study, however, study findings are routinely fedback to the study community.

Statistical Analysis

All data were analysed using SPSS. Univariate logistic regression analyses were conducted to determine the bivariate relationship between food insecurity and demographic and psychosocial predictors. Odds ratios (ORs) with p-values were calculated to determine the strength of these associations. A hierarchical multivariate logistic regression analysis was conducted to independently evaluate IPV exposure and trauma/stress on food insecurity prior to the addition of mental health risk factors, while controlling for demographic variables. Block 1 included community, maternal income, and maternal education. Block 2 included recent experiences of emotional, physical, and sexual IPV as well as maternal childhood trauma and stressful life events. Finally, Block 3 added depression and psychological distress. To determine whether depression played a mediating role on the relationship between IPV and food insecurity, meditational analyses were conducted using PROCESS macro.[45] Model number 4 was used and indirect effects were bootstrapped using 1000 samples. Beta coefficients and standard errors are reported for all paths and 95% confidence intervals are reported for the indirect effects. Models were conducted for the full sample and then for each community individually; models were split by community because of the socioeconomic, cultural, clinical and psychosocial differences between the two communities that could have significant bearings on the results of the mediation models. This process was replicated for depression as a mediator of the relationship between childhood trauma and food insecurity. Mediation models controlled for community, maternal income, maternal education, social grants, number of children in the household and HIV status; childhood trauma was controlled for in all IPV mediation models and emotional, physical, and sexual IPV was controlled for in the childhood trauma mediation models.

Results

A total of 1225 pregnant women were enrolled between March 2012 and February 2015; of these, 992 women had complete data and were included in the analysis. Missing data resulted from non-attendance at the second antenatal visit where psychosocial data was collected. A sensitivity analysis was therefore only done on sociodemographic variables (clinic, education, income, employment, social grants and whether married); those mothers included in the present analysis versus the whole cohort differed significantly only regarding whether mothers received social grants (Supplementary Table 1). Detailed baseline demographic characteristics, stratified by recruitment site, are presented in Table 1. The median age of participants was 26.6 years [standard deviation (SD) 5.8]. The sample was characterized by low SES - 77% of mothers had a monthly income of less than R1,000 (approximately 100 USD), 49% of mothers were receiving social assistance, 26% reported being employed and 38% completed secondary education (high school). A minority of mothers (40%) were married or with a partner. Food security, HIV prevalence and SES quartiles were significantly different between clinics as were the majority of psychosocial variables. Households in Mbekweni were much more likely to be food insecure than households at TC Newman (45.7% versus 12.6%). Mothers at TC Newman were significantly more likely to have experienced emotional and sexual past-year IPV as well as childhood trauma and stressful life events. Co-occurrence of mental health issues was prevalent, though more so at TC Newman. Overall,

For peer review only - http://bmjopen.bmj.com/site/about/guidelines.xhtml

12% of mothers had both depression and IPV, 13% depression and childhood trauma and 16% childhood trauma and any form of IPV.

Table 1. Maternal demographic and psychological variables

	Overall n (%)	TC Newman n (%)	Mbekweni n (%)	X^2	p-value
Number of mothers	992	443	549		
Mean age of mother (SD)	26.6 (5.8)	25.7 (5.4)	27.3 (5.9)	-4.543*	***
Food insecurity					
Secure	685 (69.1)	387 (87.4)	298 (54.3)	125.53	***
Insecure	307 (30.9)	56 (12.6)	251 (45.7)		
Race					
Black	548 (55)	6 (1)	542 (99)	943.05	***
Coloured	443 (45)	437 (99)	6 (1)		
SES Quartiles					
Lowest SES	258 (26)	81 (18)	177 (32)	37.27	***
Low-moderate SES	261 (26)	117 (26)	144 (26)		
Moderate-high SES	242 (24)	109 (25)	133 (24)		
Highest SES	231 (23)	136 (31)	95 (17)		
Maternal Income					
<R1,000/month	767 (77)	330 (74)	437 (80)	7.86	*
R1000-R5000/month	212 (21)	103 (23)	109 (20)		
R5000-R10,000/month	12 (1)	9 (2)	3 (1)		
Receive social assistance	491 (49)	221 (50)	270 (49)	0.085	0.798
Maternal education					
Some secondary	613 (62)	266 (60)	347 (63)	1.037	0.308
Completed Secondary	379 (38)	177 (40)	202 (37)		
Median number of children in household	1	1	1	22.191	**
Married/cohabiting	399 (40)	200 (45)	199 (36)	10.064	*
Employed	254 (26)	132 (30)	122 (22)	7.439	**
Maternal HIV	216 (22)	17 (4)	199 (36)	151.195	***
Psychosocial risk factors					
Past year IPV					
Emotional IPV	266 (27)	155 (35)	111 (20)	27.26	***
Physical IPV	216 (22)	106 (24)	110 (20)	2.18	0.14
Sexual IPV	68 (7)	49 (11)	19 (3)	22.179	***
Probable Depression (EPDS ≥ 13)	242 (24)	112 (25)	130 (24)	0.341	0.559
Childhood trauma	335 (34)	179 (40)	156 (28)	15.761	***
Psychological Distress	208 (21)	109 (25)	99 (18)	6.39	0.011
Stressful Life Events	449 (45)	265 (60)	184 (34)	68.467	***
Co-occurrence of psychosocial risk factors					
Depression & any IPV	122 (12)	68 (15)	54 (10)	6.911	**
Depression & childhood trauma	124 (13)	76 (17)	48 (9)	15.864	***
Childhood trauma & any IPV	154 (16)	101 (23)	53 (10)	32.304	***

*p<0.05, **p<0.01, ***p<0.001; *Note: Psychological risk factors listed where above threshold; IPV above threshold = score of >1 within each subtype (mothers experiencing more than an isolated incidence in past year); Depression above threshold = score ≥ 13; Childhood trauma above threshold where score > 36; Psychological Distress dichotomized into low and high risk categories where high risk = score ≥8; Stressful life events presented where greater than 1.*

For peer review only - http://bmjopen.bmj.com/site/about/guidelines.xhtml

In bivariate analysis (Table 2), antenatal food insecurity was significantly more likely among participants from Mbekweni (aOR 5.82; 95% CI 4.20, 8.07), those who had not completed secondary school (aOR 0.43; 95% CI 0.32, 0.57), mothers with lower income levels (aOR 0.39; 95% CI 0.27, 0.56), mothers who had experienced emotional IPV (aOR 1.44; 95% CI 1.07, 1.94) or physical IPV (aOR 1.84; 95% CI 1.35,

Table 2. Hierarchical logistic regression of variables associated with food insecurity

			Block 1		Block 2		Block 3	
Variables	Unadjusted OR (95% CI)	p-value	Adjusted OR (95% CI)	p-value	Adjusted OR (95% CI)	p-value	Adjusted OR (95% CI)	p-value
Demographic variables								
Community	5.82 (4.20, 8.07)	***	6.02 (4.30, 8.41)	***	8.22 (5.60, 12.06)	***	7.85 (5.29, 11.66)	***
Maternal Income	0.39 (0.27, 0.56)	***	0.42 (0.28, 0.62)	***	0.44 (0.29, 0.66)	***	0.44 (0.29, 0.66)	***
Maternal Education	0.43 (0.32, 0.57)	***	0.42 (0.31, 0.58)	***	0.45 (0.32, 0.63)	***	0.46 (0.33, 0.64)	***
Intimate Partner Violence								
Emotional IPV	1.44 (1.07, 1.94)	*			1.67 (1.09, 2.56)	*	1.60 (1.04, 2.46)	*
Physical IPV	1.84 (1.35, 2.52)	***			1.41 (0.91, 2.18)	0.121	1.32 (0.85, 2.05)	0.216
Sexual IPV	1.62 (0.98, 2.68)	0.061			1.77 (0.92, 3.39)	0.085	1.50 (0.78, 2.89)	0.253
Trauma/Stress+								
Childhood Trauma	1.49 (1.13, 1.97)	**			1.66 (1.18, 2.33)	**	1.52 (1.08, 2.15)	*
Stressful Life Events	0.96 (0.90, 1.02)	0.157			0.98 (0.91, 1.06)	0.585	0.93 (0.86, 1.01)	0.089
Mental Health+								
Depression (EPDS)	1.09 (1.06, 1.12)	***					1.05 (1.01, 1.08)	**
Psychological Distress	1.04 (1.01, 1.08)	*					1.05 (1.00, 1.10)	0.080
			Block X^2(df)	p-value	Block X^2(df)	p-value	Block X^2(df)	p-value
			188.93 (3)	***	8.44 (2)	*	15.75 (2)	***

*p<0.05, **p<0.01, ***p<0.001
+ Trauma/Stress and Mental Health variables were included as continuous scores in regression analyses.

2.52) in the past twelve months and mothers with higher levels of antenatal depression (aOR 1.09; 95% CI 1.06, 1.12), childhood trauma (aOR 1.49; 95% CI 1.13, 1.97) and psychological distress (aOR 1.04; 95% CI 1.01, 1.08).

Hierarchical regression

A hierarchical logistic regression was done to investigate the additive impact of risk factor groups on food security (Table 2). Throughout all blocks, community, maternal education and maternal income remained significantly associated with food insecurity. In block 2 among IPV, trauma and stress risk factors, adjusting for maternal sociodemographic factors and community, emotional IPV and childhood trauma were significant predictors of food insecurity. In the final model (block 3), which incorporated all psychological variables and demographic variables, mothers from Mbekweni were almost eight times (aOR 7.85; 95% CI 5.29, 11.66) as likely as TC Newman mothers to experience antenatal food insecurity. Mothers who completed secondary school were 54% less likely to experience food insecurity (aOR 0.46; 95% CI 0.33, 0.64) compared to mothers who did not complete secondary school. Similarly, mothers with higher incomes were 56% (aOR 0.44; 95% CI 0.29, 0.67) less likely to experience food insecurity.

For peer review only - http://bmjopen.bmj.com/site/about/guidelines.xhtml

Mothers who experienced emotional IPV in the past twelve months were 60% more likely (aOR 1.60; 95% CI 1.04, 2.46), mothers with higher depression scores on EPDS were 5% more likely (aOR 1.05; 95% CI 1.01, 1.08) and mothers with a history of childhood trauma were 52% more likely (aOR 1.52; 95% CI 1.08, 2.15) than mothers without these psychological risk factors to experience food insecurity.

Depression as a mediator of the relationship between IPV and food insecurity

In mediation models including both communities (figure 2a), depression partially mediated the relationship between emotional IPV and food insecurity (direct effect p-value = 0.0001; indirect effect = 0.16, 95% CI 0.07, 0.29) and partially mediated the relationship between physical IPV and food insecurity (direct effect p-value=0.001; indirect effect = 0.17, 95% CI 0.07, 0.28). Sexual IPV was not tested in a mediation model because the bivariate relationship between sexual IPV and food insecurity was not significant (OR 1.62; p=0.06). Mediation models were split by community due to the high significance of recruitment community as an independent predictor of food insecurity; when split by community differing degrees of mediation were found. At TC Newman (figure 2b), depression fully mediated the relationship between emotional IPV and food insecurity (direct effect p-value = 0.13; indirect effect = 0.24, 95% CI 0.05, 0.50) as well as between physical IPV and food insecurity (indirect effect = 0.23, 95% CI 0.06, 0.46). At Mbekweni (figure 2c), depression partially mediated the relationship between emotional IPV and food insecurity (direct effect p-value = 0.0004; indirect effect = 0.11, 95% CI 0.03, 0.25) and partially mediated the relationship between physical IPV and food insecurity (direct effect p-value = 0.008; indirect effect = 0.13, 95% CI 0.04, 0.29).

Depression as a mediator of the relationship between childhood trauma and food insecurity

In mediation models including both communities (figure 3), depression partially mediated the relationship between childhood trauma and food insecurity (direct effect p-value = 0.025; indirect effect = 0.13, 95% CI 0.05, 0.23). These mediation models were also split by community, due to the high significance of community as a risk factor for food insecurity. When applying mediation models to childhood trauma at TC Newman, depression did not mediate the relationship between childhood trauma and food insecurity (direct effect p-value = 0.0009; indirect effect 0.12, 95% CI -0.12, 0.35). Additionally, at Mbekweni, depression did not mediate the relationship between childhood trauma and food insecurity (direct effect p-value = 0.63; indirect effect 0.04, 95% CI -0.02, 0.13).

Discussion

Our goal was to investigate the association between IPV or maternal childhood trauma and food insecurity during pregnancy, as well as to investigate maternal depression as a mediator for these relationships in a LMIC country, South Africa. We found significant effects of emotional IPV and maternal childhood trauma on antenatal food insecurity, after adjusting for community, maternal income and education. Mothers experiencing emotional IPV or with a history of childhood trauma were 60% and 52% more likely, respectively, to live in food insecure households while pregnant. Though previous studies have investigated links between IPV or childhood trauma and food insecurity, the current study extends this research to a low resource setting with a large sample size.

Hernandez and colleagues found that IPV was a significant predictor of food insecurity and that this was mediated by depression.[24] However, this US-based study did not find significant associations between subtypes of IPV and food insecurity - only a composite measure of IPV was found to be significant. Our

For peer review only - http://bmjopen.bmj.com/site/about/guidelines.xhtml

research found that sub-types of IPV were differentially associated with food insecurity, with emotional IPV the only significant predictor in the final model. While mediation models split by site found a mediational effect of depression on this relationship at both clinics; emotional IPV did maintain a direct effect on food insecurity at Mbekweni. This may be an important distinction when planning effective interventions that consider community contexts; qualitative research has found that women feel emotionally abusive acts are more devastating than physical violence.[46] Emotional IPV, therefore, may be a critical and often overlooked risk factor for food insecurity. Further, emotional IPV may manifest differently in a LMIC setting, compared to a high-income setting, where traditional gender norms may affect women's sense of power and identity and therefore, compounded by potential mental health sequelae, may further decrease her ability to manage household resources.[47]

Maternal childhood trauma also emerged as a critical risk factor for food insecurity during pregnancy. Sun and colleagues investigated this link in a large US-based study.[22] These authors reported that childhood trauma was linked to food insecurity during pregnancy, and found that maternal depression modified this relationship. They found a dose-response relationship between number of childhood adverse events and severity of food insecurity; when considered together with depression there was a greater impact on food insecurity. While other high-income country studies have also investigated this association, these have been limited by small sample sizes (n=44, n=31).[48,49] To our knowledge, our study is the first to investigate the link between childhood adversity and food security in a LMIC or mental health as a mediator in this relationship. Childhood trauma measured by family instability, violence exposure at a young age, food insecurity in childhood and neglect is associated with many of the known risk factors of current food insecurity such as lower levels of education, employment and poor mental health outcomes in adulthood.[50] This highlights a critical link between childhood experiences and adult outcomes and the intergenerational effects of trauma. In the context of maternal mental health, this may be particularly relevant as maternal hardship and stress may increase the likelihood of a traumatic childhood for their offspring. As noted by Sun and colleagues, there is an intergenerational transmission of disadvantage, which highlights the need for a multi-faceted approach to address food insecurity.[22] Our findings reveal important intergenerational associations between food insecurity and maternal childhood exposure to violence and suggest that future research is needed to understand how intergenerational transmission of trauma occurs between mothers and children and what can be done to break this cycle. In high-prevalence settings in particular, intervention programs should offer more than nutrition support and should include trauma-informed mental health services to reduce the transmission of trauma from one generation to the next. Though further study is needed to determine if trauma counselling or interventions may help to alleviate the prevalence of food insecurity.

The co-occurrence of psychosocial risk factors was high (12-16%) in our study sample. In order to better understand how these risk factors influence one another, we investigated depression as a mediator in these relationships. In overall models depression partially mediated the relationship between emotional and physical IPV and food insecurity. Notably, the degree to which depression mediated this relationship differed between clinic communities. Depression fully mediated the relationship between emotional and physical IPV and food insecurity at TC Newman, but only partial mediation was found at Mbekweni. This highlights depression as important in the pathway through which IPV affects food insecurity at TC Newman. However, at Mbekweni, though depression also exacerbates this relationship, there may be other factors that explain the significant relationship between IPV and food insecurity. While maternal

For peer review only - http://bmjopen.bmj.com/site/about/guidelines.xhtml

income was controlled for in mediation models, SES quartiles indicate that Mbekweni mothers are economically worse off than TC Newman mothers. This may be impeding the process of food management at Mbekweni, especially in the context of IPV. In overall models investigating depression as a mediator for the relationship between childhood trauma and food security, partial mediation was again seen; however, no mediation was found when models were split by community. At TC Newman, childhood trauma maintained a significant direct effect on food insecurity. It may be that social support networks are more robust at Mbekweni, thus mitigating the downstream effects of childhood trauma in that community. A study in a similar community in South Africa found that social support buffers the effect of trauma on depression symptoms;[51] further, social support has been found to be particularly important for females, compared to males in mitigating mental health outcomes such as depression.[52] Additional research is needed to understand how or why depression mediated the effects of childhood trauma on food insecurity in overall models whereas this effect did not persist when models were split by community.

Additionally, this study indicates that community level factors should be considered when developing nutritional and mental health interventions. Many communities in South Africa are still dealing with the long-term effects of apartheid, this may have a continued effect on stress and mental health in these communities.[53] However, racial disparities exist globally affecting physical and mental health in specific communities differentially to others.[54,55] Specifically, in targeting mental health, contextual factors such as differences in stigma to accessing care, gender norms affecting agency or education levels for women, may have significant differential effects within communities. This community context may be important to understand how to best address key risk factors for food insecurity and to inform design of effective interventions.

Strengths & Limitations

The inclusion criteria for the parent study were broad to ensure generalizability. However, recruitment was done during antenatal care visits, so mothers who did not present for antenatal care or who presented in their third trimester were excluded, which may affect overall generalizability. Further, generalizability may be limited to similar population groups, specifically pregnant mothers and similar communities. In addition, approximately 200 mothers who were enrolled in the study were not included in the analysis due to incomplete data. While it is possible that this subset of mothers is at higher risk for many of the factors investigated; there were not significant differences in key factors investigated for mothers included versus those excluded in the current study. As the current study included cross-sectional data, we cannot assert the direction of causality. While a strength of this study was its investigation of the individual impact of different subtypes of IPV, too few mothers experienced or reported sexual IPV to allow this to be investigated fully. Finally, as all variables used were based on self-report, mothers may have underreported both risk factors and food security due to social desirability bias. Despite these limitations, the current study provides a novel quantitative analysis with a large sample size conducted in a LMIC. Our findings corroborate previous research on risk factors for food insecurity; and build on unpublished data in this cohort by investigating subtypes of IPV as well as maternal depression as a mediator for both childhood trauma and IPV.

Conclusions

Addressing depression during pregnancy through screening and referral services may help to alleviate the negative impact of IPV, childhood trauma and depression on food security; though direction of causality

For peer review only - http://bmjopen.bmj.com/site/about/guidelines.xhtml

cannot be asserted by the current study, significant associations between these variables and food insecurity were found. Both IPV victimization and experiencing childhood trauma were associated with depressive symptoms in mothers, after controlling for maternal income and education. This may impact their household managerial skills by decreasing motivation to obtain food, to find and hold employment and through decreased physical and cognitive functioning. Our findings highlight the importance of comprehensive programs aimed at nutrition support or food security, as well as the importance of addressing multiple concurrent psychosocial risk factors which may help to reduce food insecurity and alleviate its negative impact on child health. However, notably, mediation models indicate that the effects of maternal mental health issues are experienced differently at the two communities in this cohort highlighting the importance of program and policy efforts targeted to specific community profiles. Notably, though not the focus of the current study, both maternal income and maternal education were highly correlated with food insecurity – education especially is likely an important factor cross cutting all key risk and outcome variables – promoting educational opportunities represents an important intervention to improve maternal and thus child health.

Acknowledgements

We thank the study staff in Paarl, the study data team and lab teams, the clinical and administrative staff of the Western Cape Government Health Department at Paarl Hospital and at the clinics for support of the study. We acknowledge the advice from members of the study International Advisory Board and thank our collaborators. We thank the families and children who participated in this study.

Declaration of interest

The authors have no conflicts of interest to declare.

Funding

The study was funded by the Bill and Melinda Gates Foundation [OPP 1017641] with support from the National Institutes of Health (H3Africa 1U01AI110466-01A1). Additional support for DJS, NK, WB and HJZ, and for research reported in this publication was from the South African Medical Research Council (SAMRC) and the UK Royal Society, Newton Advanced Fellowship. WB is supported by the SAMRC National Health Scholars programme; NK receives support from the SAMRC under a Self-Initiated Research Grant. The views and opinions expressed are those of the authors and do not necessarily represent the official views of the SAMRC.

Author contributions:

WB & JP conceptualized the analysis and wrote the first draft of the manuscript. HJZ is principal investigator of the parent study; DS leads the psychosocial study aspects; NK and KD are co-investigators and contributed to the study design and implementation. JP conducted the data analysis. CK, JP and DS provided critical inputs on the manuscript. All authors read and approved the final manuscript.

Data sharing statement: No additional data are available. All study instruments used in this analysis are available to requesting researchers.

For peer review only

References

1. Bickel G, Nord M, Price C, Hamilton W, Cook J, Guide to Measuring Household Food Security, Revised 2000. U.S. Department of Agriculture, Food and Nutrition Service, Alexandria VA. March, 2000.
2. FAO, IFAD, UNICEF, WFP and WHO. 2017. The State of Food Security and Nutrition in the World 2017. Building resilience for peace and food security. Rome, FAO.
3. Borders AE, Grobman WA, Amsden LB, Holl JL. Chronic stress and low birth weight neonates in a low-income population of women. *Obstet gynecol* 2007; **109**(2 Pt 1): 331-8.
4. Laraia BA, Siega-Riz AM, Gundersen C. Household food insecurity is associated with self-reported pregravid weight status, gestational weight gain, and pregnancy complications. *J Am Diet Assoc* 2010; **110**(5): 692-701.
5. Cook JT, Black M, Chilton M, et al. Are food insecurity's health impacts underestimated in the U.S. population? Marginal food security also predicts adverse health outcomes in young U.S. children and mothers. *Advances in nutrition (Bethesda, Md)* 2013; **4**(1): 51-61.
6. Cook JT, Frank DA, Levenson SM, et al. Child food insecurity increases risks posed by household food insecurity to young children's health. *J Nutr* 2006; **136**(4): 1073-6.
7. Bronte-Tinkew J, Zaslow M, Capps R, Horowitz A, McNamara M. Food insecurity works through depression, parenting, and infant feeding to influence overweight and health in toddlers. *J Nutr* 2007; **137**(9): 2160-5.
8. Cook JT, Frank DA, Berkowitz C, et al. Food insecurity is associated with adverse health outcomes among human infants and toddlers. *J Nutr* 2004; **134**(6): 1432-8.
9. Zaslow M, Bronte-Tinkew J, Capps R, Horowitz A, Moore KA, Weinstein D. Food security during infancy: implications for attachment and mental proficiency in toddlerhood. *Matern Child Health J* 2009; **13**(1): 66-80.
10. Hernandez DC, Jacknowitz A. Transient, but not persistent, adult food insecurity influences toddler development. *J Nutr* 2009; **139**(8): 1517-24.
11. Rose-Jacobs R, Black MM, Casey PH, et al. Household food insecurity: associations with at-risk infant and toddler development. *Pediatrics* 2008; **121**(1): 65-72.
12. Kirkpatrick SI, McIntyre L, Potestio ML. Child hunger and long-term adverse consequences for health. *Arch Pediatr Adoles Med* 2010; **164**(8): 754-62.
13. Ivers LC, Cullen KA. Food insecurity: special considerations for women. *Am J Clin Nutr* 2011; **94**(6): 1740S-4S.
14. Davison KM, Kaplan BJ. Food insecurity in adults with mood disorders: prevalence estimates and associations with nutritional and psychological health. *Ann Gen Psychiatry* 2015; **14**: 21.
15. Martin MS, Maddocks E, Chen Y, Gilman SE, Colman I. Food insecurity and mental illness: disproportionate impacts in the context of perceived stress and social isolation. *Pub Health* 2016.
16. Melchior M, Caspi A, Howard LM, et al. Mental health context of food insecurity: a representative cohort of families with young children. *Pediatrics* 2009; **124**(4): e564-72.
17. Sorsdahl K, Slopen N, Siefert K, Seedat S, Stein DJ, Williams DR. Household food insufficiency and mental health in South Africa. *J Epidem Community Health* 2011; **65**(5): 426-31.
18. Koen N, Wyatt GE, Williams JK, et al. Intimate partner violence: associations with low infant birthweight in a South African birth cohort. *Metab Brain Dis* 2014; **29**(2): 281-99.
19. Brittain K, Myer L, Koen N, et al. Risk Factors for Antenatal Depression and Associations with Infant Birth Outcomes: Results From a South African Birth Cohort Study. *Paediatr Perinat Epidemiol* 2015.
20. Ross J, Hanlon C, Medhin G, et al. Perinatal mental distress and infant morbidity in Ethiopia: a cohort study. *Archives of Disease in Childhood: Fetal and Neonatal Edition* 2011; **96**(1): F59-64.
21. Rahman A, Harrington R, Bunn J. Can maternal depression increase infant risk of illness and growth impairment in developing countries? *Child Care Health Dev* 2002; **28**(1): 51-6.

For peer review only - http://bmjopen.bmj.com/site/about/guidelines.xhtml

22. Sun J, Knowles M, Patel F, Frank DA, Heeren TC, Chilton M. Childhood Adversity and Adult Reports of Food Insecurity Among Households With Children. *Am J Prev Med* 2015.
23. Chilton M, Knowles M, Rabinowich J, Arnold KT. The relationship between childhood adversity and food insecurity: 'It's like a bird nesting in your head'. *Public Health Nutr* 2015; **18**(14): 2643-53.
24. Hernandez DC, Marshall A, Mineo C. Maternal depression mediates the association between intimate partner violence and food insecurity. *J Women's Health* 2014; **23**(1): 29-37.
25. Gibbs A, Corboz J, Jewkes R. Factors associated with recent intimate partner violence experience amongst currently married women in Afghanistan and health impacts of IPV: a cross sectional study. *BMC Pub Health* 2018; **18**.
26. Wehler C, Weinreb LF, Huntington N, et al. Risk and protective factors for adult and child hunger among low-income housed and homeless female-headed families. *Am J Public Health* 2004; **94**(1): 109-15.
27. Radimer KL, Olson CM, Greene JC, Campbell CC, Habicht J-P. Understanding hunger and developing indicators to assess it in women and children. *J Nutr Educ* 1992; **24**(1): 36S-44S.
28. Pellowski JA, Barnett W, Kuo CC, Koen N, Zar HJ, Stein DJ. Investigating tangible and mental resources as predictors of perceived household food insecurity during pregnancy among women in a South African birth cohort study. *Soc Sci Med* 2017; **187**: 76-84.
29. Statistics South Africa. (Sept 2018). *Drakenstein Municipality*. Retrieved from http://www.statssa.gov.za/?page_id=993&id=drakenstein-municipality.
30. Zar HJ, Barnett W, Myer L, Stein DJ, Nicol MP. Investigating the early-life determinants of illness in Africa: the Drakenstein Child Health Study. *Thorax* 2015; **70**(6): 592-4.
31. Stein DJ, Koen N, Donald KA, et al. Investigating the psychosocial determinants of child health in Africa: The Drakenstein Child Health Study. *J Neurosci Meth* 2015.
32. Jewkes R. Intimate partner violence: causes and prevention. *Lancet (London, England)* 2002; **359**(9315): 1423-9.
33. Shamu S, Abrahams N, Temmerman M, Musekiwa A, Zarowsky C. A systematic review of African studies on intimate partner violence against pregnant women: prevalence and risk factors. *PloS one* 2011; **6**(3): e17591.
34. Cox JL, Holden JM, Sagovsky R. Detection of postnatal depression. Development of the 10-item Edinburgh Postnatal Depression Scale. *Br J Psychiatry* 1987; **150**: 782-6.
35. Lawrie TA, Hofmeyr GJ, de Jager M, Berk M. Validation of the Edinburgh Postnatal Depression Scale on a cohort of South African women. *S Afr Med J* 1998; **88**(10): 1340-4.
36. Tsai AC, Scott JA, Hung KJ, et al. Reliability and validity of instruments for assessing perinatal depression in African settings: systematic review and meta-analysis. *PLoS One* 2013; **8**(12): e82521.
37. Bernstein DP, Fink L, Handelsman L, et al. Initial reliability and validity of a new retrospective measure of child abuse and neglect. *Am J Psych* 1994; **151**(8): 1132-6.
38. Bernstein D, Fink L. Childhood Trauma Questionnaire: a retrospective self-report. San Antonio: The Psychological Corporation; 1998.
39. Myer L, Stein DJ, Grimsrud A, Seedat S, Williams DR. Social determinants of psychological distress in a nationally-representative sample of South African adults. *Soc Sci Med (1982)* 2008; **66**(8): 1828-40.
40. Beusenberg M, Orley J. A user's guide to the self reporting questionnaire (SRQ). Division of Mental Health. Geneva: World Health Organization: 1994.
41. Ventevogel P, De Vries G, Scholte WF, et al. Properties of the Hopkins Symptom Checklist-25 (HSCL-25) and the Self-Reporting Questionnaire (SRQ-20) as screening instruments used in primary care in Afghanistan. *J Soc Psychiatry* 2007; **42**(4): 328-35.
42. Harpham T, Reichenheim M, Oser R, et al. Measuring mental health in a cost-effective manner. *Health Policy Plan* 2003; **18**(3): 344-9.

For peer review only - http://bmjopen.bmj.com/site/about/guidelines.xhtml

43. Hadley C, Lindstrom D, Tessema F, Belachew T. Gender Bias in the Food Insecurity Experience of Ethiopian Adolescents. *Soc Sci Med (1982)* 2008; **66**(2): 427-38.
44. Leonard WR. Household-level strategies for protecting children from seasonal food scarcity. *Soc Sci Med (1982)* 1991; **33**(10): 1127-33.
45. Hayes AF. Introduction to Mediation, Moderation, and Conditional Process Analysis: A Regression-Based Approach. *Guilford Press* 2013.
46. World Health Organization. WHO multi-country study on women's health and domestic violence against women: summary report of initial results on prevalence, health outcomes and women's responses. Geneva, WHO, 2012.
47. Department of Women, Children & People with Disabilities (DWCPD). Stop violence against women in South Africa. Cape Town, DWCPD, 2013.
48. Chilton MM, Rabinowich JR, Woolf NH. Very low food security in the USA is linked with exposure to violence. *Public Health Nutr* 2014; **17**(1): 73-82.
49. Chilton M, Knowles M, Bloom SL. The Intergenerational Circumstances of Household Food Insecurity and Adversity. *J Hunger Environ Nutr* 2017; **12**(2): 269-97.
50. Messina N, Grella C. Childhood trauma and women's health outcomes in a California prison population. *Am J Pub Health* 2006; **96**(10): 1842-8.
51. Casale M, Wild L, Cluver L, Kuo C. Social support as a protective factor for depression among women caring for children in HIV-endemic South Africa. *J Behav Med* 2015; **38**(1): 17-27.
52. Kendler KS, Myers J, Prescott CA. Sex differences in the relationship between social support and risk for major depression: a longitudinal study of opposite-sex twin pairs. *Am J Psychiatry* 2005; **162**(2): 250-6.
53. Jackson PB, Williams DR, Stein DJ, Herman A, Williams SL, Redmond DL. Race and Psychological Distress: The South African Stress and Health Study. *J Health Soc Behav* 2010; **51**(4): 458-77.
54. Penner LA, Hagiwara N, Eggly S, Gaertner SL, Albrecht TL, Dovidio JF. Racial Healthcare Disparities: A Social Psychological Analysis. *Eur Rev Soc Psych* 2013; **24**(1): 70-122.
55. Flores G, Lin H. Trends in racial/ethnic disparities in medical and oral health, access to care, and use of services in US children: has anything changed over the years? *Int J Equity Health* 2013; **12**: 10.

Figure legends

Figure 1. Conceptual framework for study.

Fig 2a-2c. Covariates included in the model are: community, maternal income, maternal education, social grants, number of children in the household, HIV status and childhood trauma.

Fig 3. Mediation models for both communities, for TC Newman and for Mbekweni investigating depression as a mediator for childhood trauma and food insecurity. Depression **partially** mediated the overall relationship between childhood trauma and food insecurity, but **did not mediate** the relationship at TC Newman and **did not** mediate the relationship at Mbekweni. Covariates included in the model are: community, maternal income, maternal education, social grants, number of children in the household, HIV status and emotional, physical and sexual forms of IPV.

For peer review only

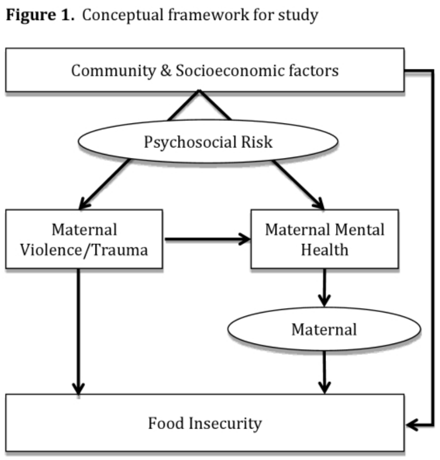

Figure 1. Conceptual framework for study.

209x297mm (300 x 300 DPI)

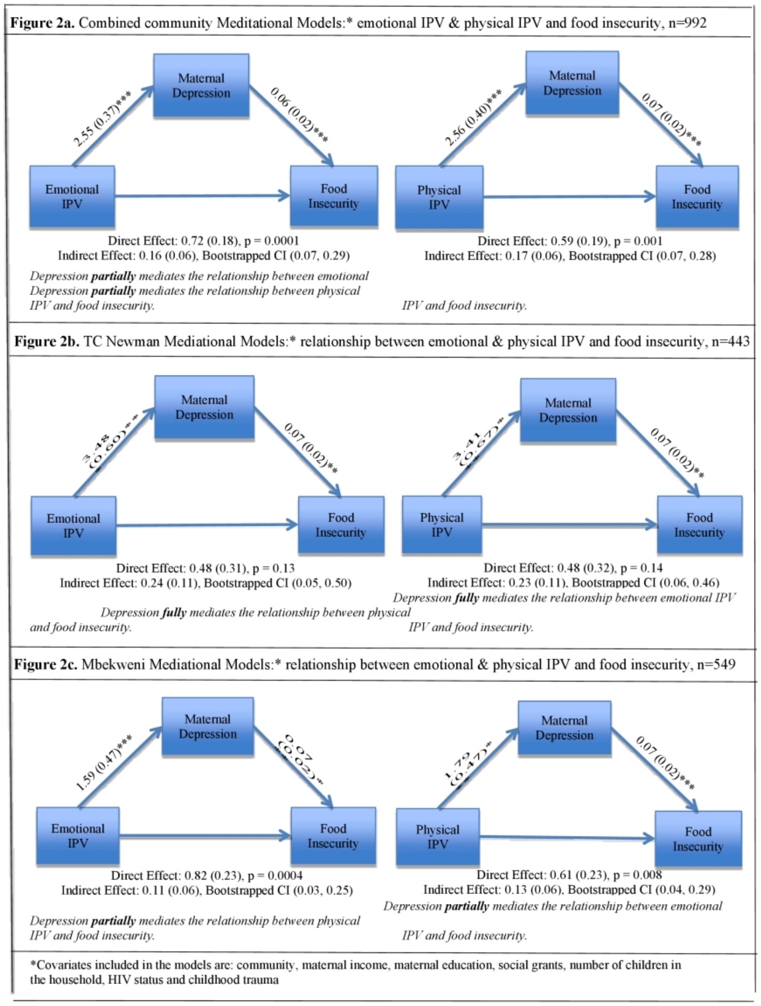

Fig 2a-2c. Covariates included in the model are: community, maternal income, maternal education, social grants, number of children in the household, HIV status and childhood trauma.

209x297mm (300 x 300 DPI)

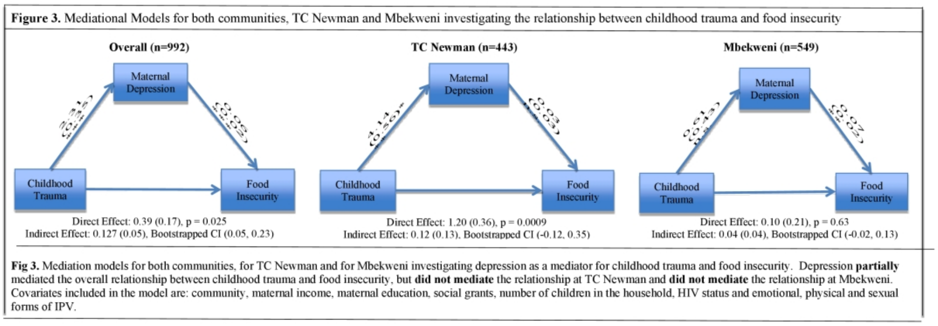

Fig 3. Mediation models for both communities, for TC Newman and for Mbekweni investigating depression as a mediator for childhood trauma and food insecurity. Depression partially mediated the overall relationship between childhood trauma and food insecurity, but did not mediate the relationship at TC Newman and did not mediate the relationship at Mbekweni. Covariates included in the model are: community, maternal income, maternal education, social grants, number of children in the household, HIV status and emotional, physical and sexual forms of IPV.

297x209mm (300 x 300 DPI)

Supplemental Table 1. Sensitivity analysis: comparison of key sociodemographic variables between participants included in and excluded from the current study.

	**Included sample**	**Excluded sample**	**p-value**
Number of mothers	992	233	
Clinic			
Mbekweni	549 (55)	131 (56)	
TC Newman	443 (45)	102 (44)	0.808
Race			
Black	548 (55)	132 (57)	
Coloured	443 (45)	101 (43)	0.708
Maternal Income			
<R1,000/month	767 (77)	168 (78)	
R1000-R5000/month	212 (21)	44 (20)	0.954
R5000-R10,000/month	12 (1)	3 (1)	
Receive social assistance	491 (49)	92 (40)	0.005
Maternal education			
Some secondary	613 (62)	127 (55)	
Completed Secondary	379 (38)	103 (45)	0.066
Median number of children in household	1	1	0.911
Married/cohabiting	399 (40)	92 (40)	0.828
Employed	254 (26)	67 (29)	0.429

For peer review only

STROBE Statement

Checklist of items that should be included in reports of observational studies

Section/Topic	Item No	Recommendation	Reported on Page No
**Title and abstract**	1	(*a*) Indicate the study's design with a commonly used term in the title or the abstract	2
		(*b*) Provide in the abstract an informative and balanced summary of what was done and what was found	2
**Introduction**			
Background/rationale	2	Explain the scientific background and rationale for the investigation being reported	3
Objectives	3	State specific objectives, including any prespecified hypotheses	3
**Methods**			
Study design	4	Present key elements of study design early in the paper	4
Setting	5	Describe the setting, locations, and relevant dates, including periods of recruitment, exposure, follow-up, and data collection	4
Participants	6	*Cross-sectional study*—Give the eligibility criteria, and the sources and methods of selection of participants	4,5
Variables	7	Clearly define all outcomes, exposures, predictors, potential confounders, and effect modifiers. Give diagnostic criteria, if applicable	4,5
Data sources/measurement	8*	For each variable of interest, give sources of data and details of methods of assessment (measurement). Describe comparability of assessment methods if there is more than one group	4,5
Bias	9	Describe any efforts to address potential sources of bias	10
Study size	10	Explain how the study size was arrived at	5
Quantitative variables	11	Explain how quantitative variables were handled in the analyses. If applicable, describe which groupings were chosen and why	4,5
Statistical methods	12	(*a*) Describe all statistical methods, including those used to control for confounding	5
		(*b*) Describe any methods used to examine subgroups and interactions	5,7
		(*c*) Explain how missing data were addressed	5
		(*d*) *Cohort study*—If applicable, explain how loss to follow-up was addressed<br>*Case-control study*—If applicable, explain how matching of cases and controls was addressed<br>*Cross-sectional study*—If applicable, describe analytical methods taking account of sampling strategy	n/a
		(*e*) Describe any sensitivity analyses	10

For peer review only - http://bmjopen.bmj.com/site/about/guidelines.xhtml

Section/Topic	Item No	Recommendation	Reported on Page No
**Results**			
Participants	13*	(a) Report numbers of individuals at each stage of study—eg numbers potentially eligible, examined for eligibility, confirmed eligible, included in the study, completing follow-up, and analysed	5
		(b) Give reasons for non-participation at each stage	
		(c) Consider use of a flow diagram	n/a
Descriptive data	14*	(a) Give characteristics of study participants (eg demographic, clinical, social) and information on exposures and potential confounders	6
		(b) Indicate number of participants with missing data for each variable of interest	5
		(c) *Cohort study*—Summarise follow-up time (eg, average and total amount)	n/a
Outcome data	15*	*Cohort study*—Report numbers of outcome events or summary measures over time	n/a
		Case-control study—Report numbers in each exposure category, or summary measures of exposure	
		Cross-sectional study—Report numbers of outcome events or summary measures	6
Main results	16	(*a*) Give unadjusted estimates and, if applicable, confounder-adjusted estimates and their precision (eg, 95% confidence interval). Make clear which confounders were adjusted for and why they were included	7
		(*b*) Report category boundaries when continuous variables were categorized	6
		(*c*) If relevant, consider translating estimates of relative risk into absolute risk for a meaningful time period	n/a
Other analyses	17	Report other analyses done—eg analyses of subgroups and interactions, and sensitivity analyses	8,10
**Discussion**			
Key results	18	Summarise key results with reference to study objectives	8
Limitations	19	Discuss limitations of the study, taking into account sources of potential bias or imprecision. Discuss both direction and magnitude of any potential bias	10
Interpretation	20	Give a cautious overall interpretation of results considering objectives, limitations, multiplicity of analyses, results from similar studies, and other relevant evidence	8,9,10
Generalisability	21	Discuss the generalisability (external validity) of the study results	10
**Other Information**			
Funding	22	Give the source of funding and the role of the funders for the present study and, if applicable, for the original study on which the present article is based	10

**Give information separately for cases and controls in case-control studies and, if applicable, for exposed and unexposed groups in cohort and cross-sectional studies.*

Note: An Explanation and Elaboration article discusses each checklist item and gives methodological background and published examples of transparent reporting. The STROBE checklist is best used in conjunction with this article (freely available on the Web sites of PLoS Medicine at http://www.plosmedicine.org/, Annals of Internal Medicine at http://www.annals.org/, and Epidemiology at http://www.epidem.com/). Information on the STROBE Initiative is available at www.strobe-statement.org.

For peer review only - http://bmjopen.bmj.com/site/about/guidelines.xhtml
